# Exploring the Spectrum of VEGF Inhibitors’ Toxicities from Systemic to Intra-Vitreal Usage in Medical Practice

**DOI:** 10.3390/cancers16020350

**Published:** 2024-01-13

**Authors:** Mariachiara Santorsola, Maurizio Capuozzo, Guglielmo Nasti, Francesco Sabbatino, Annabella Di Mauro, Giordana Di Mauro, Gianluca Vanni, Piera Maiolino, Marco Correra, Vincenza Granata, Oreste Gualillo, Massimiliano Berretta, Alessandro Ottaiano

**Affiliations:** 1 Istituto Nazionale Tumori di Napoli, IRCCS “G. Pascale”, via M. Semmola, 80131 Naples, Italy; mariachiara.santorsola@istitutotumori.na.it (M.S.); g.nasti@istitutotumori.na.it (G.N.); annabella.dimauro@istitutotumori.na.it (A.D.M.); p.maiolino@istitutotumori.na.it (P.M.); m.correra@istitutotumori.na.it (M.C.); v.granata@istitutotumori.na.it (V.G.); 2 Coordinamento Farmaceutico, ASL-Naples-3, 80056 Ercolano, Italy; m.capuozzo@aslnapoli3sud.it; 3 Oncology Unit, Department of Medicine, Surgery and Dentistry, University of Salerno, 84081 Salerno, Italy; fsabbatino@unisa.it; 4 Department of Human Pathology “G. Barresi”, University of Messina, 98125 Messina, Italy; giordana.di.mauro@hotmail.it; 5 Breast Unit, Department of Surgical Science, PTV Policlinico Tor Vergata University, 00133 Rome, Italy; gianluca.vanni@ptvonline.it; 6 SERGAS (Servizo Galego de Saude), NEIRID Laboratory (Neuroendocrine Interactions in Rheumatology and Inflammatory Diseases), IDIS (Instituto de Investigación Sanitaria de Santiago), Research Laboratory 9, Santiago University Clinical Hospital, 15706 Santiago de Compostela, Spain; oreste.gualillo@sergas.es; 7 Department of Clinical and Experimental Medicine, University of Messina, Via Consolare Valeria, 98125 Messina, Italy

**Keywords:** vascular endothelial growth factor, vascular endothelial growth factor inhibitor, toxicity, monoclonal antibodies, receptor tyrosine kinase inhibitor

## Abstract

**Simple Summary:**

Vascular Endothelial Growth Factor inhibitors (VEGFi), commonly employed for diverse medical conditions such as advanced neoplasms and ocular diseases, pose potential adverse effects. This review delineates the principal VEGFi utilized in clinical practice and investigates the causes, recognition, management, and prevention of VEGFi toxicities. Its aim is to assist oncologists in both clinical practice and the design of clinical trials.

**Abstract:**

The use of Vascular Endothelial Growth Factor inhibitors (VEGFi) has become prevalent in the field of medicine, given the high incidence of various pathological conditions necessitating VEGF inhibition within the general population. These conditions encompass a range of advanced neoplasms, such as colorectal cancer, non-small cell lung cancer, renal cancer, ovarian cancer, and others, along with ocular diseases. The utilization of VEGFi is not without potential risks and adverse effects, requiring healthcare providers to be well-prepared for identification and management. VEGFi can be broadly categorized into two groups: antibodies or chimeric proteins that specifically target VEGF (bevacizumab, ramucirumab, aflibercept, ranibizumab, and brolucizumab) and non-selective and selective small molecules (sunitinib, sorafenib, cabozantinib, lenvatinib, regorafenib, etc.) designed to impede intracellular signaling of the VEGF receptor (RTKi, receptor tyrosine kinase inhibitors). The presentation and mechanisms of adverse effects resulting from VEGFi depend primarily on this distinction and the route of drug administration (systemic or intra-vitreal). This review provides a thorough examination of the causes, recognition, management, and preventive strategies for VEGFi toxicities with the goal of offering support to oncologists in both clinical practice and the design of clinical trials.

## 1. Introduction

The Vascular Endothelial Growth Factor (VEGF) family comprises several secreted glycoproteins, including VEGF-A (commonly referred to as “VEGF”), Placenta Growth Factor (PlGF), VEGF-B, VEGF-C, and VEGF-D [1,2]. Additionally, VEGF-E genes are located within viral genomes [3], and VEGF-F is a component of snake venom, although their functions in humans remain poorly understood [1,2,3]. Endocrine gland-derived vascular endothelial growth factor (EG-VEGF) is primarily expressed in adrenal glands, testes, placenta, and ovaries, where the VEGF receptor level correlates both with macroscopic residual diseases and the risk of disease progression [4,5]. VEGFs exert their effects based on their varying affinities for VEGF receptors, namely, VEGFR-1, VEGFR-2, and VEGFR-3. They belong to the receptor tyrosine kinase (RTK) superfamily, exhibiting intrinsic intracellular tyrosine kinase activity upon ligand binding to transmit signals to cells [6,7].

VEGFs are constitutively produced by immune and stromal cells and can be induced by hypoxia in various cell types. VEGFR-1 and VEGFR-2 are expressed on vascular endothelial cells, while VEGFR-3 is particularly expressed on lymphatic endothelial cells [8]. Upon the binding of VEGFs to their receptors, particularly on the surface of endothelial cells, receptor dimerization occurs, bringing two receptor molecules together. This dimerization initiates the auto-phosphorylation of specific tyrosine residues within the intracellular domain of the receptor. Phosphorylated tyrosine residues then serve as docking sites for various intracellular signaling proteins. Among the primary downstream pathways activated, Src kinase, focal adhesion kinase (FAK), the phosphoinositide 3-kinase (PI3K)/Akt pathway, and the mitogen-activated protein kinase (MAPK) pathway are notable [6,7,8]. These pathways play a crucial role in endothelial cell proliferation and migration and the synthesis of pro-angiogenic factors.

VEGFs play a pivotal role in regulating the growth of blood and lymphatic vessels, influencing various aspects of human physiology and pathology; notably, the pathologic and excessive “neo-angiogenesis”, the process of forming new blood vessels from existing ones, is implicated in conditions such as diabetic macular edema (DME), diabetic retinopathy (PDR), macular edema secondary to retinal vein occlusion (RVO), neovascular (wet) age-related macular degeneration (AMD), choroidal neovascularization (CNV), and cancer [3]. A new class of drugs, referred to as VEGF inhibitors (VEGFi), limits the formation of new blood vessels where excessive angiogenesis is detrimental, and they have demonstrated efficacy in these conditions.

## 2. Pharmacokinetics and Pharmacodynamic Aspects of VEGFi

Distinguishing between two classes of VEGFi, specifically antibodies/chimeric proteins and small molecules, is imperative due to their distinct mechanisms of action and consequential therapeutic implications. Antibodies and chimeric proteins operate by binding to VEGF ligands with high specificity, thereby impeding their interaction with receptors and inhibiting downstream signaling cascades. Conversely, small molecules target intracellular signaling pathways, modulating kinase activity or interfering with downstream effectors. This mechanistic dichotomy underscores the need for meticulous categorization, as it significantly shapes drug designs, therapeutic effectiveness, and the potential for adverse effects.

### 2.1. VEGFi Belonging to the Antibodies/Chimeric Proteins Category

**Figure 1:**
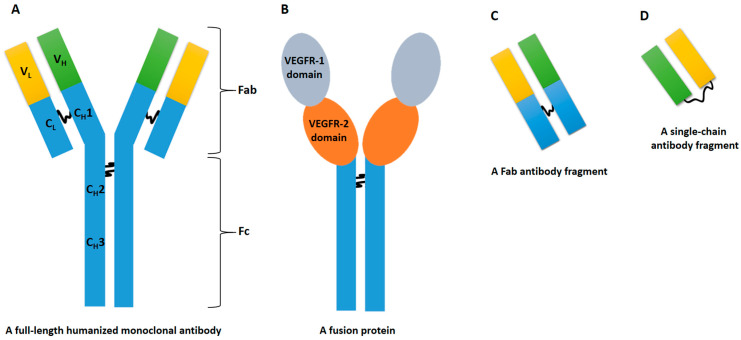
The figure delineates four distinct types of VEGFi belonging to the antibodies/chimeric proteins class, each characterized by substantial structural disparities that intricately shape their pharmacological profiles. Bevacizumab (**A**), the first type, is a full-length humanized monoclonal antibody that functions by binding to VEGF, thereby impeding its interaction with VEGF receptors. In contrast, the entity represented by (**B**) (i.e., aflibercept) adopts a fusion protein architecture, fusing VEGF receptor segments with a human IgG1 Fc fragment. This design transforms aflibercept into a soluble decoy receptor, effectively antagonizing both VEGF and placental growth factor (PlGF). Ranibizumab, denoted as (**C**), constitutes a monoclonal antibody fragment employing a specifically designed Fab fragment to selectively inhibit VEGF-A. Conversely, brolucizumab, identified by (**D**), features a single-chain antibody fragment, leveraging its diminutive size to enhance penetration into retinal tissues, thereby facilitating targeted inhibition of VEGF-A. Abbreviations elucidating key structural components are as follows: Fab (Fragment Antigen-Binding), Fc (Fragment Crystallizable), C (Constant), H (Heavy Chain), L (Light Chain), and V (Variable). Additionally, the VEGFi structural elements are underscored by VEGFR-1 domain (Vascular Endothelial Growth Factor Receptor-1 domain) and VEGFR-2 domain (Vascular Endothelial Growth Factor Receptor-2 domain).

Anti-VEGF-based drugs play crucial roles in the treatment of ocular and oncological pathologies by targeting VEGF and modulating angiogenesis. Their distinct molecular characteristics, pharmacokinetics, and mechanisms of action enable them to address a range of medical conditions with varying efficacies and safety profiles. The most used are bevacizumab, ramucirumab, aflibercept, brolucizumab, and ranibizumab (Figure 1).

Bevacizumab is a humanized immunoglobulin G1 (IgG1) monoclonal antibody with a molecular weight (MW) of 149 kDa [9,10]. It is primarily employed in the treatment of metastatic cancers, including colorectal, breast, cervical, non-squamous non-small cell lung cancer (NSCLC), ovarian, fallopian tube, or glioblastoma, primary peritoneal cancer, renal cell carcinoma, and hepatocellular carcinoma [11]. Bevacizumab also exhibits off-label applications in ophthalmology. The catabolism occurs thorough proteolysis, involving nonspecific elimination pathways and target-mediated elimination by VEGF-expressing cells [12]. Hence, like all the other anti-VEGF drugs, it does not affect the activity of drug-metabolizing enzymes in the liver. It binds to VEGF-A and has a half-life of approximately 20 days after systemic administration [13]. Notably, bevacizumab has been explored for off-label indications, such as angiosarcoma, gliomas, malignant pleural mesothelioma, medulloblastoma (pediatric), hemangiopericytoma, and malignant solitary fibrous tumor, as well as hereditary hemorrhagic telangiectasia (HHT), DME, and AMD [14,15,16].

Ramucirumab is a humanized IgG1 monoclonal antibody that binds specifically to VEGFR-2 and blocks VEGF-A, VEGF-C, and VEGF-D binding [17]. The MW is 146 kDa, which is similar to bevacizumab. It is used mainly in oncological contexts, including gatric, colorectal, non-small cell lung cancers, and hepatocarcinoma [18,19,20]. The safety of intraocular injection of ramucirumab has not yet been extensively tested. However, the direct inhibition of VEGFR-2 over the inhibition of its ligands may be a more suitable therapeutic target. Its half-life after systemic administration is about 20 days [21].

Aflibercept is a recombinant fusion protein between the Fc portion of IgG1 and binding portions of VEGFR 1 and 2, with a MW of 115 kDa [22]. Its primary use is in the treatment of metastatic colorectal cancer and various ocular conditions, including neovascular (wet) AMD, RVO, DME, myopic choroidal neovascularization (mCNV), and diabetic retinopathy (DR) [23]. Aflibercept binds to VEGF-A, VEGF-B, and PlGF and has a half-life of approximately 6 days after systemic administration and 7.13 days after intra-vitreal injection. It undergoes catabolism predominantly through proteolysis [24].

Brolucizumab, a single-chain antibody fragment Fv (scFv) with an MW of 26 kDa, targets all isoforms of VEGF-A [25]. Its primary use is in treating AMD and DME [26]. While it is still under investigation for oncological applications, brolucizumab has catabolism similar to aflibercept, involving proteolysis. It has a relatively short serum half-life of approximately 5.6 h after systemic administration and 4.5 days after intra-vitreal delivery, suggesting fast systemic clearance and minimal systemic exposure [27]. Furthermore, it has been shown to have a higher binding affinity to VEGF-A isoforms than bevacizumab or ranibizumab [28].

Ranibizumab is a fragment of a recombinant, humanized IgG1 monoclonal antibody Fab with a MW of 48 kDa [29]. It is indicated for the treatment of neovascular AMD and macular edema following RVO, DME, DR, and CNV. Ranibizumab lacks the Fc region of an antibody, potentially reducing the risk of intraocular inflammation following intra-vitreal injection [30]. Its mechanism of action is similar to brolucizumab, binding to and inhibiting all the biologically active forms of VEGF-A. Ranibizumab has a half-life of approximately 3.59 days in the serum and 9 days intra-vitreally [31].

### 2.2. VEGFi Belonging to the Small Molecules Category

**Figure 2:**
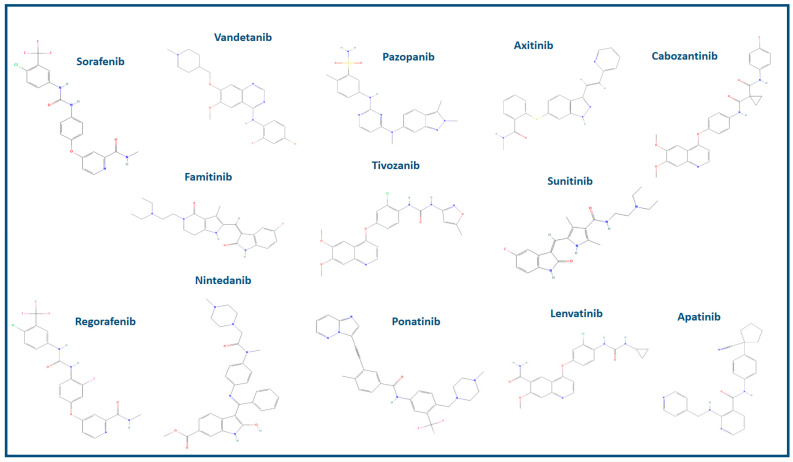
Two-dimensional chemical structures of the VEGF inhibitors mentioned in the manuscript are provided. For a comprehensive analysis of compound compositions, the “PubChem Compound” free tool (www.ncbi.nlm.nih.gov/pccompound/, accessed on 2 November 2023), an integral component of the online research resources provided by the National Center for Biotechnology Information (NCBI), is publicly accessible.

These small molecules are administered orally and are highly bioavailable. They primarily target neo-angiogenesis by acting on VEGFR-2 [32]. In fact, the binding of VEGFA to VEGFR-2 triggers significant tyrosine phosphorylation and leads to a robust angiogenic response. In contrast, VEGFR-1 exhibits only weak tyrosine kinase activity and appears to modulate angiogenesis in the capacity of a decoy receptor [33]. These small molecules are classified as multi-receptor tyrosine kinase inhibitors (RTKi), signifying their ability to inhibit tyrosine kinases associated with various receptors, each with different affinities [34,35,36,37]. In contrast to antibodies and chimeric proteins, they undergo extensive metabolism in the liver, primarily through a pathway involving hepatic cytochrome P450 enzymes. The metabolites formed are generally less pharmacologically active than the parent compounds. Importantly, the variations in the inhibition of various kinases (non-selectivity) may account for diverse activity profiles in the pathologies for which they are employed, contributing to distinct toxicity spectra, although a common type of toxicity appears to be prevalent. Here is a non-exhaustive list and an exposition of the functional features and clinical applications of the most commonly used drugs, primarily in oncological clinical settings (Figure 2). Some of these drugs have already received FDA approval, while others are currently under investigation.

Sunitinib, with a MW of approximately 398 daltons (Da), has received FDA approval for its application in the field of oncology, specifically for the treatment of renal cell carcinoma, gastrointestinal stromal tumors (GISTs), and pancreatic neuroendocrine tumors (PNET). Notably, Sunitinib has been found to inhibit multiple kinases, including VEGFR-1, VEGFR-2, VEGFR-3, c-KIT, FLT3 kinase, colony-stimulating factor 1 receptor, and RET kinase [38]. Sorafenib, with a MW of roughly 464 Da, is predominantly employed in the treatment of advanced renal cell carcinoma and hepatocellular carcinoma. It is a tyrosine kinase inhibitor that primarily targets Raf kinase and VEGFR-2. It also exhibits activity against several other kinases, including VEGFR-1, VEGFR-3, PDGFR-beta, Flt-3, and c-KIT [39]. Vandetanib, with a MW of approximately 474 Da, has received FDA approval for the treatment of medullary thyroid cancer. It inhibits VEGFR-2, VEGFR-3, and EGFR [40]. Pazopanib (MW: 437 Da) is used in the treatment of advanced renal cell carcinoma and soft tissue sarcoma. Pazopanib inhibits kinases such as VEGFR-1, VEGFR-2, VEGFR-3, c-KIT, and PDGFR [41,42]. Axitinib, with a MW of roughly 386 Da, is primarily indicated for the treatment of advanced renal cell carcinoma. It primarily inhibits VEGFR-1, VEGFR-2, and VEGFR-3 [43]. Cabozantinib, with a MW of approximately 501.63 Da, is applied in the treatment of advanced renal cell carcinoma, hepatocellular carcinoma, and medullary thyroid cancer. It inhibits VEGFR-2, c-MET, and AXL [44]. Regorafenib (MW of approximately 482 Da) is indicated for the treatment of colorectal cancer, gastrointestinal stromal tumors (GISTs), and hepatocellular carcinoma. Regorafenib inhibits a range of kinases, including VEGFR-1, VEGFR-2, VEGFR-3, c-KIT, RET, RAF-1, and BRAF [45,46,47]. Nintedanib, classified as a triple angiokinase inhibitor, boasts a MW of around 539 Da. It primarily inhibits kinases like VEGFR, FGFR, and PDGFR. While it primarily serves in the treatment of idiopathic pulmonary fibrosis, the FDA has endorsed its use in non-small cell lung cancer in oncological contexts [48,49]. Ponatinib, with a MW of approximately 569 Da, is primarily used for the treatment of chronic myeloid leukemia (CML). It is a potent tyrosine kinase inhibitor that targets a range of kinases, including BCR-ABL, which is associated with CML, as well as kinases such as VEGFR-2, SRC, KIT, and PDGFR [50]. Lenvatinib, characterized by an MW of approximately 426.88 Da, is a tyrosine kinase inhibitor applied in the treatment of differentiated thyroid cancer and hepatocellular carcinoma, with FDA approval for these specific oncological indications. Lenvatinib inhibits VEGFR-1, VEGFR-2, VEGFR-3, FGFR, RET, and KIT kinases [51,52]. Tivozanib, with an MW of roughly 454 Da, recently received FDA approval for the treatment of advanced renal cell carcinoma. It primarily inhibits VEGFR-1, VEGFR-2, and VEGFR-3 [53]. Famitinib is an innovative and powerful multi-targeted TKI that primarily targets VEGFR-2. However, it also exhibits low affinity for PDGFR and c-kit-associated kinases. Currently, it is the subject of clinical trials for cancer treatment, including renal cell carcinoma. Its MW is approximately 419 Da [54]. Apatinib is undergoing investigation for the treatment of advanced gastric cancer and various other malignancies. It is a highly selective inhibitor of VEGFR-2, with a MW of about 494 Da [55,56].

In conclusion, all these small molecule VEGFR-2 inhibitors belong to the class of RTKi and are predominantly indicated for the treatment of specific oncological conditions. Notably, Nintedanib is also used for a non-oncological indication in idiopathic pulmonary fibrosis.

## 3. Etiology and Epidemiology of VEGFi Toxicity

**Figure 3:**
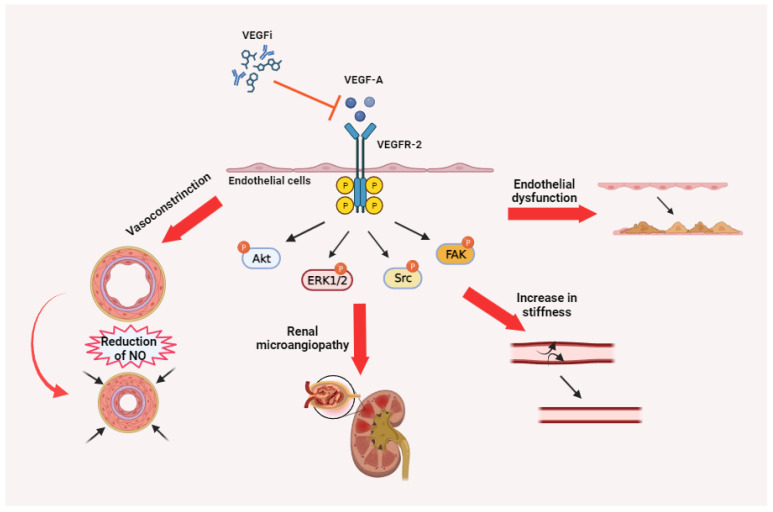
Synthesis of the etiology of toxicities attributable to VEGFi. Particularly relevant is the impact of the alteration of the VEGFR-2/VEGF-A pathway on endothelial cell function at multiple anatomical and functional levels.

The toxicities observed with VEGFi in clinical practice stem from the disruption of the physiological functions of the VEGF/VEGFR pathway, particularly involving VEGF-A/VEGFR-2 (Figure 3).

The clinical manifestation of toxicities induced by various VEGFi drugs is linked to their varying affinity for VEGFA and VEGFR2 [57], as well as the consequences of inhibiting other receptors (e.g., in the case of VEGFi RTKi, the inhibition of hERG channels expressed in the heart) [58]. The primary form of toxicity associated with VEGFi is vascular toxicity. However, vascular and microvascular damage is also associated with a reduction in mucosal trophism and the physiological microenvironment of mucosae, primarily in the intestinal mucosa. In summary, all VEGFi drugs can interfere with

- Regulation of blood vessel tone by affecting the production of nitric oxide, a molecule that plays a crucial role in regulating blood vessel dilation [59,60,61].
- Microvessel perfusion and microvascular density lead to a reduction in arterioles and capillaries. This reduction can result in increased peripheral resistance [62,63].
- Physiological survival and proliferation of endothelial cells make existing blood vessels more fragile and susceptible to damage [64].
- Renewal of endothelial cells in response to (micro)trauma, which exposes subendothelial collagen and activates the coagulation cascade [65,66].
- Permeability is controlled by the endothelial cells of glomerular capillaries in the kidneys [67].
- Tissue repair and wound healing [68,69].

VEGFi toxicity assessment is primarily based on the Common Terminology Criteria for Adverse Events (CTCAE) (accessible at https://ctep.cancer.gov/protocoldevelopment/electronic_applications/ctc.htm, accessed on 5 December 2023), providing valuable insights into this form of toxicity. The incidence is reported for all grades in Table 1 [70,71,72,73,74,75,76,77,78,79,80,81].

## 4. Pathophysiology of VEGFi Toxicity

In this section, we delineate the underlying mechanisms of each clinically significant toxicity. This detailed elucidation is crucial for a comprehensive understanding of the adverse effects associated with the treatment. By delineating these mechanisms, our objective is to offer insights into the biological processes and pathways involved, thereby facilitating subsequent comprehension, recognition, and clinical management. Hypertension: Caused by the disruption of the VEGF/VEGFR pathway, particularly involving VEGF-A/VEGFR-2, endothelial dysfunction plays a crucial role in vascular toxicity, specifically hypertension, in the clinical context. Blood pressure homeostasis relies on the regulation of blood vessel relaxation and constriction. VEGFi can disturb this equilibrium, leading to vasoconstriction and elevated blood pressure [59,60,61]. Proteinuria: The inhibition of VEGF-A leads to alterations in glomerular permeability, which can present as proteinuria and peripheral edema [82]. Thromboembolic events: These events involve the development of blood clots within a blood vessel due to endothelial damage or dysfunction. These clots have the potential to dislodge and traverse the circulatory system, leading to embolism and the subsequent obstruction of blood flow. Most commonly, this phenomenon presents as either deep vein thrombosis or pulmonary embolism [65,83]. Cardiac toxicity: VEGF is involved in protecting cardiomyocytes (heart muscle cells) from injury and promoting their survival [84,85]. Therefore, inhibiting this pathway can result in damage to cardiomyocytes and a decline in cardiac function. However, the direct effects on the heart are more closely associated with cumulative dosages, reflecting prolonged exposure to VEGFi. In most cases, the heart is indirectly impacted, primarily due to complications related to alterations in blood pressure, increased capillary permeability, and the formation of thromboemboli. These events indirectly impair cardiac function by compromising blood flow and oxygen delivery to the heart. In the case of VEGFi belonging to the class of RTKi, direct and variable inhibition of hERG (human Ether-à-go-go-Related Gene) channels can lead to changes in cardiac electrical conduction [86,87]. These membrane channels are responsible for the movement of potassium ions, specifically the rapid delayed rectifier potassium current (Ikr), crucial in the repolarization phase of the cardiac action potential. When hERG channels are affected, it can lead to a prolongation of the cardiac action potential, particularly the QT interval. This prolonged QT interval can predispose individuals to Torsades de Pointes (TdP), a type of ventricular arrhythmia that can potentially escalate to life-threatening ventricular fibrillation and sudden cardiac death [88,89]. Hemorrhage: The inhibition of VEGF can compromise blood vessel integrity and induce abnormal vascular permeability in the presence of micro- and macro-traumas, ultimately resulting in bleeding [77]. Bowel and nasal septum perforation: These serious events can arise from VEGFi-induced tumor necrosis, which weakens the mucosal wall, or from VEGFi-induced injury with impaired wound healing, as well as arteriolar thrombosis leading to regional ischemia and subsequent perforation [90,91,92]. Skin toxicity: The precise mechanism underlying VEGFi-induced skin toxicity remains incompletely understood, but it is hypothesized to stem from the disruption of VEGF signaling pathways, leading to alterations in blood flow and vascular permeability in the skin [93]. Reversible Posterior Leukoencephalopathy Syndrome (RPLS): The underlying mechanism of RPLS is not fully understood but is likely attributed to the disruption of the VEGF pathway and an increase in vascular permeability at the blood-brain barrier level [94]. Infusion-related hypersensitivity reactions: The exact mechanism causing infusion-related reactions remains unclear, despite the use of humanized monoclonal antibodies with a lower risk of immunogenicity compared to chimeric monoclonal antibodies, as anti-VEGF agents [95]. Hypothyroidism: The mechanisms underlying VEGFi-induced hypothyroidism may involve direct cytotoxic effects on thyroid follicular cells and vascular alterations in the thyroid gland [92]. Asthenia and fatigue: As these adverse events predominantly manifest in cancer patients, establishing a definitive cause—whether solely attributable to the medication, the cancer itself, or other associated treatments—poses a challenge. Asthenia and fatigue associated with VEGFi may stem from various effects, such as hypothyroidism, myocardial changes, VEGF-inhibitor-induced anorexia, and dehydration resulting from diarrhea [96]. Gastrointestinal (GI) toxicity: VEGF inhibition can diminish blood flow to mucosal tissues and enhance vascular permeability, resulting in the leakage of fluid and proteins into surrounding tissues, thereby contributing to inflammation and damage [71,97]. Anorexia: Even in the absence of gastrointestinal (GI) toxicity, it may still occur. In these cases, it is hypothesized to involve intricate interactions within the central nervous system, potentially associated with changes in neuroendocrine signaling and appetite regulation [98]. Myelotoxicity: VEGF plays a pivotal role in sustaining the microenvironment of the bone marrow, exerting influence over hematopoiesis. Myelotoxicity associated with VEGFi can be attributed to at least two distinct effects. Firstly, the inhibition of VEGF can disrupt the vascular network within the bone marrow, consequently compromising the essential blood supply required for the proliferation and survival of hematopoietic cells [99]. This effect is observed commonly with both antibodies and RTKi. Secondly, there is evidence suggesting that VEGFi may directly impact hematopoietic stem cells and progenitor cells by interfering with their normal differentiation, resulting in diminished blood cell production [100,101]. It is noteworthy that many VEGFi RTKi may also inhibit KIT, FLT3, and PDGF receptors expressed on hematopoietic progenitor cells, playing roles in their growth and differentiation.

Conjunctival hemorrhage, vitreous floaters, rhegmatogenous retinal detachments, and retinal hemorrhage are primarily attributed to mechanical damage during the injection process. Ocular Hypertension: Anti-VEGF agents are typically administered in volumes ranging from 0.05 to 0.1 mL, leading to a physiologic increase in intraocular pressure (IOP) to 30–50 mm Hg due to the sudden volume change. However, this acute elevation is transient, with IOP returning to baseline within 1 h. Notably, repetitive intra-vitreal anti-VEGF injections have been linked to persistent ocular hypertension (IOP measurements >25 mm Hg post-injection), necessitating continuous IOP-lowering therapy. Proposed mechanisms for this chronic condition include microparticle obstruction of the trabecular meshwork and direct effects on trabecular meshwork cells induced by intra-vitreal VEGFi [102,103]. Intraocular inflammation: The pathogenesis remains unclear. It has been postulated that it may be linked to patient-specific immune responses, manufacturing impurities, and errors in provider preparation [80]. Brolucizumab-Associated Retinal Vasculitis (BARV): The most plausible hypotheses involve severely reduced vascular perfusion, particularly in susceptible eyes, especially those with diminished baseline retinal blood flow. Another hypothesis is the local production of anti-brolucizumab antibodies. Vasculitis may affect arteries, veins, and capillaries, with large and small retinal arteries demonstrating various combinations of narrowing, occlusion, and perivascular sheathing. Signs of retinal ischemia encompass whitening, cotton wool spots, intraretinal hemorrhage, and pericentral acute middle maculopathy [104]. Infectious endophthalmitis: It is an infection affecting the internal structures of the eye, leading to inflammation and tissue destruction [105]. A summary of the likely mechanisms responsible for toxic effects is provided in Table 2.

## 5. Clinical Identification of VEGFi Toxicity

Hypertension: Hypertension stands out as the most prevalent adverse event associated with VEGFi treatment. According to the American College of Cardiology (https://www.acc.org/Guidelines, accessed on 18 November 2023), it represents a consistent rise in systemic arterial pressure, characterized by a blood pressure reading exceeding 140/90 mm Hg, or 130/80 mm Hg in patients with underlying chronic kidney disease or diabetes. Specifically, VEGFi-induced hypertension in individuals without pre-existing hypertension is defined as a sustained increase (lasting ≥24 h) in systolic pressure (>140 mm Hg) or diastolic pressure (>90 mm Hg) from the initiation of medical intervention [106]. In patients with pre-existing hypertension, it is characterized by a symptomatic increase of more than 20 mm Hg (diastolic) or reaching levels greater than 140/90 mm Hg, necessitating an adjustment in the baseline medical treatment. Acute clinical manifestations of hypertension may include headaches, dizziness, visual disturbances, epistaxis, and conjunctival or retinal hemorrhages. Furthermore, individuals may experience fatigue, shortness of breath, and cognitive impairment. The gradual and chronic elevation of blood pressure is typically asymptomatic but contributes to vascular damage, thereby amplifying the risk of cardiovascular events such as stroke, myocardial infarction, and heart failure [59,60,61]. Proteinuria: Proteinuria may manifest as peripheral edema, foamy or frothy urine, and hypertension [82]. Thromboembolic events: Signs and symptoms of thromboembolic events are leg swelling and pain, shortness of breath, chest discomfort, atypical lung sounds, and coughing up blood. Cardiac toxicity: Early signs and symptoms may include asthenia and fatigue, shortness of breath, and fluid retention, with manifestations such as peripheral edema and hepatomegaly. To determine the staging of heart failure, please refer to the guidelines provided by the American College of Cardiology (https://www.acc.org/Guidelines, accessed on 18 November 2023). QT prolongation may present as asthenia and unexplained syncopal episodes. Hemorrhage: Hemorrhage, or bleeding, is a recognized adverse event associated with the use of VEGFi, although life-threatening events are very rare. The clinical presentation of hemorrhage is diverse, ranging from epistaxis to severe gastrointestinal bleeding (melena). Hemoptysis and hematuria occur more frequently in oncology patients with lung and renal cancers. Therefore, in these cases, it can be challenging to attribute these signs solely to VEGFi or disease progression. Intracranial hemorrhage is a more severe and potentially life-threatening effect described in both primary and secondary tumor localizations [77]. Bowel and nasal septum perforation: The perforation of the intestinal wall presents with acute abdominal pain, peritonitis, fever, nausea, vomiting, constipation, or diarrhea, along with distension of the abdomen. Shock, characterized by low blood pressure and a rapid heart rate, may occur later in the course of the condition. Fever (>37.5 °C) and leukocytosis are frequently present but may be masked by the anergizing effect of any chemotherapy that may be administered. The nasal septum perforations manifest with various signs and symptoms, including epistaxis, difficulty breathing through the nose, whistling or hissing noises during breathing, particularly when inhaling or exhaling, nasal crusting, alteration in the external appearance of the nose, dryness and irritation, and pain [90,91,92]. Skin toxicity: Skin toxicity is a prevalent occurrence observed in patients undergoing treatment with VEGFi belonging to the RTKi class. The primary skin toxicities include hand–foot syndrome (HFS) and rash. Additionally, a diverse array of dermatologic adverse events may be encountered, such as mucositis, pruritus, alopecia, seborrheic dermatitis-like rash, xerosis, and subungual hemorrhage [107]. The severity of these manifestations can vary and is commonly graded according to the CTCAE. RPLS: The most common clinical symptoms include severe headache, nausea, confusion, cortical blindness, and seizures [94]. Infusion-related hypersensitivity reactions: These reactions encompass a spectrum from allergic symptoms to anaphylactoid reactions and include manifestations such as flushing, itching, hypertension, wheezing, rigors, chest pain, and diaphoresis. Hypothyroidism: Signs and symptoms of hypothyroidism are often nonspecific and may encompass fatigue, unexplained weight gain, heightened sensitivity to cold, dry skin and hair with potential hair loss, muscle aches and weakness, joint pain, constipation, and depressive symptoms. Women may additionally encounter menstrual irregularities, and hoarseness in the voice may be observed [92]. Asthenia and fatigue: Asthenia and fatigue characterize a condition marked by reduced energy levels and physical or mental weariness, primarily relying on subjective clinical reports. Asthenia entails a widespread perception of diminished strength or weakness, affecting both physical and mental dimensions. Conversely, fatigue is characterized by an intense sensation of tiredness and exhaustion [108]. Gastrointestinal (GI) toxicity: Nausea and vomiting manifest discernible signs in a physical examination, including heightened salivation, pallor, and a general appearance of unease. Subjective expressions of nausea by patients often precede the occurrence of vomiting. In contrast, mucositis becomes evident through the inflammation and ulceration of mucous membranes, particularly observable in the oral cavity, where redness, swelling, and ulcerative lesions may occur. Patients report accompanying symptoms such as pain, discomfort, and difficulty in swallowing. Notably, oral mucositis can lead to the additional symptom of altered taste sensation, known as dysgeusia. In the case of diarrhea, physical examination reveals heightened bowel sounds, abdominal cramping, and urgency. Crucial indicators include the consistency and frequency of stools. Patients frequently describe symptoms encompassing frequent, loose, or watery stools, often coupled with abdominal pain, bloating, and fatigue. Anorexia: In cases of anorexia, the physical examination may reveal unintentional weight loss, muscle wasting, and a reduction in subcutaneous fat. Signs of malnutrition may also be present. Patients describe a lack of appetite and reduced food intake, and the consequences of anorexia extend to nutritional deficiencies, weakness, and an overall decline in functional status. Myelotoxicity: Anemia, marked by a shortage of red blood cells or hemoglobin, frequently manifests with symptoms such as fatigue, weakness, pale skin, and shortness of breath. Neutropenia and lymphopenia, indicating a reduction in neutrophils and lymphocytes, respectively, can impair the immune system, heightening susceptibility to infections. Thrombocytopenia, characterized by a low platelet count, may result in easy bruising, prolonged bleeding, and the development of petechiae. The severity of myelotoxicity can vary and is commonly evaluated according to the CTCAE. Conjunctival hemorrhage: This toxicity is characterized by a visible red discoloration in the conjunctiva. It manifests as subconjunctival bleeding, which is observable on the eye’s surface. Patients may experience an irritation or a sensation of a foreign body in the eye. Vitreous floaters: Vitreous floaters present as perceived specks, dots, or cobweb-like shapes in the visual field. These floaters move with eye movements and are generally asymptomatic. However, they can cause visual disturbances, impacting the overall quality of vision. Rhegmatogenous retinal detachments: The onset of rhegmatogenous retinal detachments is often sudden, marked by flashes of light (photopsia) and the appearance of new or increased floaters. Patients may describe a shadow or curtain descending across their visual field, accompanied by a decrease in visual acuity. Retinal hemorrhage: Retinal hemorrhage is characterized by the presence of blood within the retina. This can lead to sudden changes in vision, with the extent and location of the hemorrhage determining the severity of visual impairment. Patients may report pain or discomfort associated with this toxicity. Ocular hypertension: Ocular hypertension may endure without producing discernible discomfort or alterations in vision. Consequently, routine eye examinations are imperative for the identification and surveillance of ocular hypertension, particularly during VEGFi administration. This condition serves as a risk factor for the onset of glaucoma, distinguished by optic nerve damage and the loss of the visual field. Intraocular inflammation: Referred to as pseudoendophthalmitis, it manifests as acute-onset intraocular inflammation devoid of infection. Typically, symptoms arise between 24 h and seven days post-injection. Common manifestations include blurred vision, floaters, pain, and photophobia. The primary symptoms are often blurred vision and floaters, with pain occurring in only up to 46% of patients. Photophobia is infrequent. While visual acuity decreases from baseline at presentation, it frequently returns to pre-injection levels following the resolution of inflammation. BARV: Establishing a diagnosis is possible for patients who have received intra-vitreal brolucizumab in the last 8 weeks, showing clinical evidence of inflammation in the absence of infectious endophthalmitis. Furthermore, visual acuity is consistently worse than the acuity at the time of the BARV [109]. Infectious endophthalmitis: Symptoms typically manifest between one and six days following the inciting injection. Every patient experiences decreased vision and pain, with the latter being severe in endophthalmitis and mild-to-moderate in intraocular inflammation and BARV. Conjunctival injection, anterior chamber cell with hypopyon, and vitritis are commonly observed during examination. Table 3 presents the description of primary clinical manifestations associated with VEGFi toxicities.

## 6. Assessment of Toxicities Induced by VEGFi: Pragmatic Approaches for Intervention

Hypertension: It is imperative to promptly identify any increase in patients’ blood pressure during VEGFi therapy using a sphygmomanometer. Regular blood pressure assessments both before and after VEGFi treatment are recommended [110]. Patients should be educated on self-monitoring their blood pressure at home by conducting measurements at least twice daily. Blood pressure monitoring through a Holter device is recommended in cases where highly suspected signs and/or symptoms of elevated blood pressure persist despite normal values in single assessments. Proteinuria: Pathologic proteinuria is characterized by the excretion of more than 150 mg of protein per day in a comprehensive urinalysis test (or ≥2+ at urinalysis dipstick). In such instances, additional evaluation with a 24 h urine collection for protein is recommended. When administering VEGFi, it is essential to regularly assess urinary protein excretion to monitor potential proteinuria [111]. Thromboembolic events: Patients should be educated to recognize signs and symptoms of thromboembolic events, such as leg swelling and pain, shortness of breath, chest discomfort, atypical lung sounds, and coughing up blood. Patients should be strongly encouraged to promptly report any unusual symptoms. Healthcare providers should consider employing imaging techniques such as doppler ultrasound for the evaluation of deep vein thrombosis or computed tomography pulmonary angiography for the diagnosis of pulmonary embolism (PE) when clinical suspicion arises. Monitoring relevant laboratory parameters, including D-dimer levels, which may be elevated in the presence of thromboembolic events, is advisable. An increase in D-dimer levels should trigger further investigation [112]. Cardiac toxicity: To mitigate the risk of cardiac arrhythmias, it is crucial to periodically monitor the QT interval when administering VEGFi RTKi. Rigorous cardiac monitoring during VEGFi administration is vital for early detection of potential cardiac toxicity. This monitoring protocol should encompass regular assessments of cardiac function through echocardiography, specifically for monitoring ejection fraction, along with routine electrocardiograms (ECG) [72,113]. Hemorrhage: Monitoring for bleeding events during VEGFi therapy is essential and should involve regular clinical assessments to detect signs like nosebleeds, gastrointestinal symptoms, or neurological changes. Additionally, hematological tests are necessary to monitor platelet counts, coagulation parameters, and hemoglobin levels.

Bowel and nasal septum perforation: Patients, particularly those with identifiable risk factors, should undergo vigilant clinical monitoring for the early detection of signs and symptoms associated with bowel or nasal septum perforation. Skin toxicity: Periodic clinical inspection is crucial for identifying early signs of cutaneous toxicity and monitoring it. RPLS: After clinical suspicion (sudden headache and confusion) of RPLS, crucial diagnostic tests include brain imaging, typically conducted through magnetic resonance imaging (MRI). This imaging modality reveals edema in the white matter of the posterior regions of the cerebral hemispheres. Additionally, blood pressure monitoring is essential, as RPLS is frequently associated with hypertension. Infusion-related hypersensitivity reactions: Close clinical observation is crucial during the initiation of drug infusion and for at least one hour following its completion to detect infusion-related hypersensitivity reactions. Hypothyroidism: It is imperative to regularly monitor blood biochemistry, including thyroid function, at least on a monthly basis, during VEGFi treatment to promptly detect and address hypothyroidism. Asthenia and fatigue: Asthenia and fatigue are diagnosed through systematic and periodic clinical examinations, complemented by in-depth discussions with the patient and the administration of specific questionnaires to assess fatigue. Special attention is dedicated to identifying potential underlying causes, including chronic illnesses, medications, or psychological factors. Laboratory tests, such as blood tests assessing factors like complete blood count and thyroid function, may be conducted to rule out potential physiological causes. Additionally, the diagnostic process involves a thorough review of the patient’s lifestyle, sleep patterns, and stress levels [114]. Gastrointestinal (GI) toxicity: It is primarily diagnosed through clinical examination and anamnestic collection. Assessment tests of electrolytes in clinical biochemistry can indicate the severity of vomiting and/or diarrhea (loss of potassium and/or sodium, increase in chloride, acid-base balance disturbances) [97].

Anorexia: The diagnosis of anorexia involves a clinical assessment, encompassing an evaluation of body weight, measurement of body mass index (BMI), blood tests to assess nutritional parameters including glycemia, lipids, albuminemia, hemoglobin, thyroid function, and an assessment of eating habits. Additionally, psychological criteria, such as pre-existing distorted eating behaviors and anxiety related to food and body weight, are considered [115,116]. Myelotoxicity: Myelotoxicity is typically assessed through a combination of clinical evaluation and laboratory tests. Conjunctival hemorrhage: The clinical presentation, characterized by the presence of subconjunctival bleeding, is sufficient for diagnosis. Vitreous floaters: Visualization techniques like fundus examination, optical coherence tomography (OCT), or ultrasound may be used to assess the vitreous and confirm the presence of floaters. Rhegmatogenous retinal detachments: Imaging modalities such as ultrasound or optical coherence tomography (OCT) play a crucial role in diagnosing retinal detachments. These tests help visualize the detached retina and identify the presence of retinal breaks or tears [117]. Retinal Hemorrhage: Fundus examination, optical coherence tomography (OCT), or fluorescein angiography may be employed to visualize retinal hemorrhages and assess their extent and impact on retinal structures. Ocular hypertension: In the evaluation of IOP during VEGFi treatment, tonometry is a key component of eye examinations. Goldmann Applanation Tonometry (GAT) is the preferred method, considered the gold standard for accuracy [118]. Additional methods include non-contact (or air-puff) tonometry and rebound tonometry. The choice of tonometry method is influenced by factors such as the patient’s age, cooperation level, and potential variations in corneal thickness, which can impact accuracy. Visual field evaluation is most commonly conducted using Goldmann or automated perimetry methods. Intraocular inflammation: Laboratory tests such as a complete blood count provide insights into systemic inflammation, while markers like erythrocyte sedimentation rate (ESR) and C-reactive protein (CRP) offer additional information about the inflammatory status. Elevations in these markers may prompt further investigation into the presence of intraocular inflammation. Instrumental tests play a crucial role in diagnosing intraocular inflammation. Slit-lamp examinations offer a detailed view of the anterior segment, while fundus photography and OCT contribute to the assessment of posterior segment inflammation. Fluorescein angiography aids in visualizing vascular changes associated with inflammation, providing a comprehensive diagnostic approach [119]. BARV: Conducting a fundus examination and obtaining serum and vitreous cultures are essential steps to rule out infectious endophthalmitis. Infectious endophthalmitis: Laboratory tests involve needle-based vitreous sampling for microbiologic analysis, including antibiograms, to obtain ocular fluid for diagnostic purposes. Measurement of inflammatory markers such as ESR or CRP can contribute to supporting the diagnosis. Ocular imaging techniques, such as ultrasounds or OCT, may provide additional insights into the extent of inflammation and structural changes within the eye. An elevated white blood cell count may be associated with the severity of the condition [104].

A concise depiction of methods for assessing and monitoring the adverse effects of VEGFi is presented in Table 4.

## 7. Management of VEGFi Toxicities

Hypertension: Patients with pre-existing hypertension face an elevated risk of experiencing aggravated blood pressure elevation when undergoing VEGFi treatment. Other risk factors include diabetes mellitus, underlying cardiovascular disease, tobacco use, chronic kidney disease, hyperlipidemia, obesity, and advanced age [120]. To mitigate these risks, individuals should consider quitting tobacco use, reducing body weight, and addressing hyperlipidemia. In the event of hypertension arising as a result of VEGFi treatment, it can be effectively managed with antihypertensive medications, such as angiotensin-converting enzyme inhibitors or angiotensin II receptor blockers [121]. The majority of patients who receive these medications successfully achieve blood pressure control and do not necessitate additional treatments. If a patient experiences grade two or three hypertension, VEGFi treatment should be temporarily halted until blood pressure levels return to their baseline values or fall below 160/100 mm Hg. If blood pressure remains poorly controlled one month after the onset of hypertension, discontinuation of the VEGFi drug should be considered. Permanent discontinuation is also necessary in the case of hypertensive emergencies, which are defined as a diastolic blood pressure exceeding 150 mm Hg, with or without end-organ damage. Blood pressure levels must be monitored at least monthly for four to six months after discontinuing VEGFi treatment until they return to baseline values. Patients without a history of hypertension should discontinue antihypertensive drugs once their blood pressure returns to normal levels. For patients with pre-existing hypertension, ongoing therapy with antihypertensive drugs is recommended, and their blood pressure should be monitored by their primary care physician [122]. Proteinuria: Risk factors associated with proteinuria include pre-existing hypertension, underlying renal disease, renal-cell cancers, diabetes mellitus, and immunosuppression [123,124]. To prevent proteinuria, it is essential to focus on controlling blood pressure, quitting tobacco use, reducing body weight, and managing hyperlipidemia. ACE inhibitors and angiotensin II receptor blockers (ARBs) have demonstrated the potential to reduce the severity of proteinuria and the risk of renal disease progression. However, it is important to note that there are no prospective studies confirming the efficacy of these drugs in treating this adverse event, making evidence-based treatment recommendations challenging. Adjusting the VEGFi dosage can help alleviate proteinuria while maintaining the therapeutic benefit of the drug. If 24 h urine protein levels exceed 2 g, a temporary suspension of VEGFi is recommended, with resumption when levels drop below 2 g. In cases of nephrotic syndrome (24 h urine protein >3.5 g), discontinuation of treatment is advised [125,126]. Thromboembolic events: The incidence of thromboembolic events is determined by a multitude of factors, encompassing a history of thromboembolic conditions, certain types of cancers (more prevalent in ovarian, pancreatic, bone, or brain tumors as opposed to other malignancies), prior surgical interventions, increased age, cardiac or respiratory insufficiency, prolonged periods of immobilization, endocrine therapy, cytotoxic chemotherapy, and the utilization of vascular catheters. These factors are commonly encountered in patients within the field of oncology [127,128]. To reduce the risk of thromboembolism in patients undergoing treatment with VEGFi, it is recommended to explore the use of aspirin at daily dosages below 325 mg, akin to the approach taken with the general population. It is worth emphasizing that the use of aspirin does not appear to substantially elevate the risk of bleeding in VEGFi-treated patients. Nonetheless, vigilant monitoring for bleeding events remains crucial. For patients who are stable and asymptomatic, the commencement of VEGFi treatment can be considered six months after experiencing an arterial thromboembolic event. Furthermore, in cases involving patients with multiple risk factors, allergies, a history of gastrointestinal bleeding, or bleeding disorders, the use of low-molecular-weight heparin may be contemplated as an alternative to aspirin [129]. In cases of new deep venous thrombosis or pulmonary embolism (grade 3 or 4 venous thromboembolism), it is advisable to suspend VEGFi for a minimum of two weeks. Subsequently, reinitiating VEGFi therapy is a viable option once the patient is on a stable anticoagulant regimen and lacks additional risk factors (as outlined below). However, when confronted with a life-threatening venous thromboembolism (grade 4 thromboembolism), discontinuing VEGFi treatment permanently is the recommended course of action. Anticoagulation therapy with low-molecular-weight heparin or warfarin should be initiated, especially emphasizing the preference for low-molecular-weight heparin. This preference stems from the fact that the majority of patients in this clinical context are affected by cancer. Such patients tend to exhibit more significant fluctuations in their warfarin dosage, compounded by reduced compliance with therapy and increased challenges in hematological and biochemical monitoring [130]. In the event of an arterial thromboembolic occurrence while a patient is under VEGFi treatment, discontinuing the anticancer agent on a permanent basis is the recommended course of action [127,128,129,130]. Cardiac toxicity: Patients with a history of cardiovascular diseases, such as coronary artery disease, congestive heart failure, or arrhythmias, may be at greater risk of cardiac complications when using VEGFi [131]. A thorough risk-benefit assessment, conducted by both an oncologist and a cardiologist, is advisable prior to initiating therapy in high-risk patients. To mitigate risks, all individuals should cease tobacco use, manage body weight, and address hyperlipidemia. In cases exhibiting symptoms or when the left ventricular ejection fraction falls below 50%, even in asymptomatic patients, it is recommended to suspend VEGFi therapy. Subsequently, cardiac monitoring and prompt referral to a cardiologist should be undertaken [132]. In instances of cardiac toxicity, the therapeutic approach involves the administration of medications belonging to the same drug classes recommended for managing heart failure unrelated to VEGFi, as outlined in the guidelines established by the American College of Cardiology. These drug classes include beta-blockers, angiotensin-converting enzyme (ACE) inhibitors, angiotensin II receptor blockers (ARBs), and diuretics. The utilization of these medications aims to mitigate the adverse effects on cardiac function, enhance contractility, and alleviate symptoms associated with cardiac toxicity, aligning with the established protocols for heart failure management. However, for individuals with uncontrolled cardiac disease or a reduced left ventricular ejection fraction (<50%), starting VEGFi therapy is not advisable. Hemorrhage: The management of bleeding events related to VEGFi depends on the severity and location of the bleed. In milder cases, dose adjustments or temporary discontinuation of the medication may be considered, along with the administration of hemostatic agents to control bleeding. Supportive measures are necessary, and in severe cases, interventional radiology or surgical procedures may be required to address hemorrhage, depending on the site of bleeding [133,134]. Bowel and nasal septum perforation: Numerous risk factors contribute to the development of these perforations. These factors include the presence of an unresected primary tumor, chronic irritation of the nasal mucosa, prior exposure to radiotherapy, a history of peptic ulcer disease, prolonged usage of nonsteroidal anti-inflammatory drugs, diverticulosis, chemotherapy-induced colitis, and previous surgical procedures. Patients, especially those with identifiable risk factors, should undergo vigilant monitoring for early signs and symptoms of bowel or nasal septum perforation. In the suspicion of intestinal perforation, it is necessary to perform an urgent orthostasis abdominal X-ray to guide the patient toward a surgical approach within 72 h from the onset of symptoms. In cases where mucosal perforations emerge, discontinuation of VEGFi treatment is imperative [94]. Skin toxicity: Preventative measures encompass diligent skincare and moisturization. Mild grades are typically addressed with the application of topical corticosteroids, complemented by oral antihistamines to manage associated pruritus. In instances where the toxicity exceeds grade two or attains a higher severity, temporary suspension or permanent discontinuation of VEGFi may be warranted, contingent on the severity and the patient’s response to treatment. Certain specific toxicities, such as skin- and/or hair-depigmentation, generally do not require dose modifications and are reversible, although they can negatively impact quality of life and patient self-perception [135,136]. RPLS: The prompt recognition of such syndrome should immediately prompt the suspension of VEGFi. Referral of the patient to a neurologist is mandatory for accurate diagnosis and management of the syndrome, including supportive care for headaches and seizures. Infusion-related hypersensitivity reactions: The management of infusion-related reactions aligns with the approach for other hypersensitivity reactions. Immediate discontinuation of the infusion is crucial, followed by the prompt administration of antihistamines and corticosteroids. For more severe reactions, such as those with imminent respiratory collapse and circulatory collapse, epinephrine, bronchodilators, oxygen, and intra-venous fluids should be administered. If the decision is made to restart the anti-VEGF infusion, a reduced infusion rate is advisable, and premedication with corticosteroids and antihistamines should precede the initiation of the infusion. In cases of life-threatening reactions, permanent discontinuation is recommended. The involvement of the Anesthetist is mandatory both in the management of the acute phase of the reaction and in the planning of the drug rechallenge. Hypothyroidism: In response to VEGFi-induced hypothyroidism, the standard approach involves thyroid hormone replacement therapy (HRT). Levothyroxine is commonly administered to restore normal thyroid hormone levels and manage associated symptoms. The management strategy may require adjustments to dosage or even discontinuation of VEGFi treatment, depending on the severity of hypothyroidism and the success of HRT. It is imperative to regularly monitor thyroid function, at least on a monthly basis, during VEGFi treatment to promptly detect and address hypothyroidism. Clinicians should be aware of the potential thyroid-related adverse effects associated with VEGFi and consider vigilant monitoring. Timely referrals to an Endocrinologist may be necessary to ensure the optimal management of patients manifesting this adverse effect [137]. Asthenia and fatigue: The medical intervention for these symptoms should be multi-faceted and interdisciplinary. It should encompass a comprehensive approach that addresses the condition from various perspectives, combining medical and behavioral interventions. This approach typically involves prescribing medications to target specific medical issues contributing to asthenia, psychological evaluation to assess and address any psychological factors (such as depression, anxiety, or stress contributing to asthenia), nutritional support, including consultation with a Dietitian to evaluate nutritional status and provide guidance on potential supplementation, and physical rehabilitation to maintain muscle strength and overall physical well-being [138]. Gastrointestinal (GI) toxicity: From a therapeutic perspective, the management of gastrointestinal toxicity requires a targeted approach. Medications such as 5-HT3 receptor antagonists (e.g., ondansetron) can be utilized to alleviate nausea and vomiting. Antidiarrheal agents like loperamide may help manage diarrhea. Nutritional support, including dietary counseling and monitoring, is essential. Anorexia: When anorexia is not accompanied by GI toxicity, Psychiatrists may become involved to assess and manage any coexisting conditions, such as depression or anxiety. Selective serotonin reuptake inhibitors (SSRIs) or other antidepressant medications may be prescribed, particularly when there is comorbid depression or anxiety. Fluoxetine is among the SSRIs commonly utilized in the context of anorexia. However, in severe cases where anorexia significantly impacts the patient’s overall well-being and quality of life, consideration for dose adjustments or discontinuation of VEGFi therapy should be taken into account. Consultation with a Nutritionist and Psychologist for comprehensive support may prove beneficial in optimizing the therapeutic strategy. This collaborative approach ensures a holistic assessment of the patient’s needs and facilitates a well-rounded treatment plan [139]. Myelotoxicity: Although the incidence of grade >2 myelotoxicity is low with VEGFi compared to conventional chemotherapy, RTKi may induce grade 3/4 anemia (8%), neutropenia (18%), thrombocytopenia (9%), and lymphopenia (18%) [140,141]. The severity of myelotoxicity may be dose-dependent, with higher doses of VEGFi correlating with an increased likelihood and intensity of hematologic adverse events. Therefore, careful monitoring during treatment, including periodic complete blood count examinations and dosage adjustments, including potential modifications or suspensions, is imperative. Conjunctival hemorrhage, vitreous floaters, rhegmatogenous retinal detachments, and retinal hemorrhage: Some toxicities are associated with the intraocular administration method and are consistently reported with all available anti-VEGF agents, exhibiting frequencies that do not significantly vary by drug (conjunctival hemorrhage 20–40%, vitreous floaters 5–15%, rhegmatogenous retinal detachments 0.013%, retinal hemorrhage 1–10%) [142,143,144,145]. These events are reversible and can be managed solely through observation in cases of conjunctival hemorrhage and vitreous floaters or through more invasive interventions in severe instances of rhegmatogenous retinal detachments (pneumatic retinopexy, scleral buckling, and vitrectomy) and retinal hemorrhage (laser therapy and vitrectomy). The injection itself invariably represents a form of mechanical trauma and must be conducted by highly skilled personnel in an aseptic environment. The process of administering intra-vitreal VEGFi entails introducing a needle through the conjunctiva and sclera into the vitreous cavity. The penetration of the needle and the associated pressure from the injection can induce mechanical trauma to the blood vessels in the conjunctiva, potentially leading to hemorrhage. Furthermore, the precise location of an intra-vitreal injection is crucial. If the injection is too anterior, damage may occur to the crystalline lens or ciliary body. Conversely, if the injection site is too posterior, the needle may breach the vitreous base, harm the ora serrata, or even penetrate the retina. Ocular hypertension: Risk factors for chronic ocular hypertension include the absence of post-injection subconjunctival reflux, the use of smaller needles, tunneled injection techniques, a small vitreous volume indicated by a short axial length, frequent injections, and a prior diagnosis of glaucoma. Treatment options encompass the use of topical medications such as prostaglandin analogs, beta-blockers, alpha agonists, and carbonic anhydrase inhibitors. In cases where medication is inadequate or contraindicated, surgical interventions like trabeculectomy, laser trabeculoplasty, and minimally invasive glaucoma surgeries (MIGS) may be considered viable alternatives to reduce intraocular pressure. Monitoring is crucial, involving regular assessment of intraocular pressure and visual field evaluations to monitor the potential toxicity of VEGFi. Preventative measures in high-risk patients may involve the use of topical drugs such as apraclonidine, timolol, dorzolamide, brimonidine, brinzolamide, or anterior chamber paracentesis. However, the latter introduces additional risks of infection and iatrogenic injury [146,147]. Intraocular inflammation: The standard approach involves the use of topical corticosteroids. Additionally, topical antibiotics, cycloplegics, or systemic corticosteroids may be considered if there is uncertainty in the differential diagnosis with infectious endophthalmitis, as symptoms can overlap. BARV: The management of intraocular inflammation involves the use of topical corticosteroids, systemic corticosteroids, or a combination of both, guided by regular monitoring. Close collaboration between the patient and healthcare provider is integral to the management strategy, facilitating the assessment of treatment response and allowing for informed adjustments as necessary [148]. Infectious endophthalmitis: Risk factors include ectropion and the systemic use of post-injection topical antibiotics. Patient self-monitoring is essential to detect early signs of infection, declining visual acuity, and pain. Therapeutic interventions include intra-vitreal injection of antibiotics and primary pars plana vitrectomy (PPV) [149].

Attention must be directed towards pragmatic considerations and contraindications associated with the intra-vitreal administration of VEGFi. The decision to proceed with intra-vitreal administration of VEGFi should be made judiciously, and treatment resumption should be deferred until the next scheduled session under the following conditions:

- A decrease in best-corrected visual acuity (BCVA) by ≥30 letters compared to the last visual acuity assessment.
- Intraocular pressure (IOP) attaining or surpassing 30 mm Hg.
- Presence of a retinal tear.
- Incidence of rhegmatogenous retinal detachment.
- Detection of macular holes.
- Extension of retinal hemorrhage to the central fovea, or if the extent of hemorrhage is ≥50% of the total lesion area.
- Performance or planned intraocular surgery within the preceding or subsequent 28 days.

Patients exhibiting ongoing or suspected ocular or periocular infections, as well as those undergoing continuous intraocular inflammation, should be precluded from the intra-vitreal administration of VEGFi.

## 8. Reducing VEGFi-Associated Toxicities through Patient Education and Preventive Interventions

The implementation of effective deterrence and patient education strategies is paramount in mitigating the potential toxicities associated with VEGFi in clinical practice. Drawing insights from comprehensive literature reviews (referenced in this manuscript), including the guidelines established by the Italian Association of Medical Oncology (AIOM, https://www.aiom.it/linee-guida-aiom/, accessed on 29 November 2023) and the American College of Cardiology (https://www.acc.org/Guidelines, accessed on 18 November 2023), our recommendations are grounded in a thorough examination of the latest advancements and evidence-based practices in the field. Hypertension: Patients with pre-existing hypertension and those with additional risk factors, including diabetes mellitus, cardiovascular disease, and tobacco use, should be educated about lifestyle modifications. Strategies such as smoking cessation, weight reduction, and management of hyperlipidemia can contribute to lowering the risk of aggravated blood pressure elevation during VEGFi treatment. Patients should be educated on the importance of regular blood pressure monitoring and the potential need for antihypertensive medications. Recognizing the signs of hypertension and understanding the temporary suspension protocol in cases of grade two or three hypertension is essential for effective management. Proteinuria: Controlling blood pressure, quitting tobacco use, and managing body weight are crucial preventive measures for reducing the risk of proteinuria. Patient education should emphasize the importance of adhering to these lifestyle modifications. Patients need to be informed about the role of ACE inhibitors and angiotensin II receptor blockers in managing proteinuria. Understanding the threshold levels for temporary suspension of VEGFi and the considerations for discontinuation in cases of nephrotic syndrome is vital for patient compliance. Thromboembolic events: Educating patients on the risk factors associated with thromboembolic events is essential. Recommending aspirin at daily dosages below 325 mg for certain patients can be part of a preventive strategy. Patients should be aware of the signs of thromboembolism and the importance of timely reporting. Understanding the use of low-molecular-weight heparin as an alternative in high-risk cases is crucial for comprehensive patient education. Cardiac toxicity: A collaborative risk-benefit evaluation involving oncologists and cardiologists is recommended before initiating therapy in high-risk patients. Lifestyle modifications and addressing pre-existing cardiovascular conditions are essential preventive measures. Patients should be educated on the potential for cardiac toxicity and the role of medications like beta-blockers and ACE inhibitors in managing these effects. Understanding the suspension criteria in cases of reduced left ventricular ejection fraction is vital for patient safety. Hemorrhage: Patient education should emphasize the importance of immediate reporting of any signs of bleeding. Avoiding activities that may increase the risk of bleeding, such as trauma, is crucial. Additionally, patients need to understand the significance of regular monitoring for early detection. Patients should be educated on the potential need for dose adjustments or temporary discontinuation in milder cases of bleeding. Understanding the role of hemostatic agents and the possibility of interventional radiology or surgical procedures in severe cases is essential for comprehensive patient awareness. Bowel and nasal septum perforation: Patients, especially those with identifiable risk factors, should be educated on the importance of vigilant monitoring for early signs and symptoms. Lifestyle modifications, such as avoiding prolonged usage of nonsteroidal anti-inflammatory drugs, can be emphasized. Understanding the risk factors, including prior exposure to radiotherapy and chronic irritation, is crucial. Patients should be aware of the necessity for discontinuation of VEGFi treatment if mucosal breaches or perforations become evident. Skin toxicity: Patients should be educated on diligent skincare and moisturization as preventive measures. Emphasizing the importance of early recognition and reporting of skin toxicity is crucial. Recognizing the severity of skin toxicity and understanding the potential for temporary suspension or permanent discontinuation based on severity is essential. Patients should be informed that certain specific toxicities may not require dose modifications. RPLS: Educating patients on the symptoms of RPLS and the importance of immediate reporting is crucial. Lifestyle factors contributing to increased risk, if any, should be discussed. Patients need to understand that prompt suspension of VEGFi is necessary if RPLS is suspected. Referral to a neurologist for accurate diagnosis and supportive care should be emphasized. Infusion-related hypersensitivity reactions: Patient education should focus on the importance of immediate reporting of any infusion-related reactions. Pre-infusion medication strategies, including corticosteroids and antihistamines, should be discussed. Understanding the management approach for mild and severe reactions, including the involvement of an Anesthetist, is vital. Patients should be aware of the potential for permanent discontinuation in life-threatening reactions. Hypothyroidism: Educating patients on the potential for VEGFi-induced hypothyroidism and the role of thyroid hormone replacement therapy is crucial. Patients should understand the importance of regular thyroid function monitoring and adjustments to dosage or potential discontinuation based on the severity of hypothyroidism. Timely referrals to an Endocrinologist should be encouraged. Asthenia and fatigue: Lifestyle modifications, including psychological evaluation, nutritional support, and physical rehabilitation, should be discussed as preventive measures for asthenia and fatigue. Patients should be educated on the multi-faceted approach to managing asthenia, involving medications, psychological evaluation, nutritional support, and physical rehabilitation. Gastrointestinal (GI) toxicity: Patients should be educated on the use of medications like 5-HT3 receptor antagonists for alleviating nausea and antidiarrheal agents for managing diarrhea. Understanding the targeted approach to managing GI toxicity and the importance of nutritional support is crucial. Patients need to be aware of the potential need for dose adjustments. Anorexia: Psychiatric evaluation and the potential use of SSRIs for coexisting conditions should be discussed as preventive measures. Patients should understand the role of psychiatric intervention in managing anorexia and the possibility of dose adjustments or discontinuation in severe cases impacting overall well-being. Myelotoxicity: Regular monitoring, including complete blood count examinations, is crucial for early detection and management of myelotoxicity. Understanding the potential for dose adjustments or suspensions based on the severity of myelotoxicity is essential. Patients should be aware of the dose-dependent nature and the need for careful monitoring during treatment. Ocular complications: For patients undergoing intra-vitreal administration, preventive measures include meticulous injection techniques by skilled personnel and monitoring for early signs of complications. Patients should be educated on the risks associated with intra-vitreal injections, including potential complications like conjunctival hemorrhage and retinal detachments. Recognizing signs of ocular toxicities and the importance of regular eye examinations is essential.

## 9. Conclusions

The introduction of systemically administered VEGFi for cancer treatment has had a profound impact on the prognosis of malignancies. The assessment of VEGFi toxicities is crucial in managing patient outcomes. Adverse events such as uncontrolled hypertension, proteinuria, thromboembolic events, cardiac dysfunction, and gastrointestinal perforation can significantly affect the overall well-being of patients. Close monitoring and proactive management of these toxicities are essential to optimize the risk-benefit profile and ensure a favorable prognosis for individuals undergoing VEGFi therapy. In the field of ophthalmology, the intra-vitreal administration of VEGFi therapies has revolutionized the prognosis of retinal diseases. While these treatments contribute to improved visual acuity and slowed disease progression, ocular adverse events, including endophthalmitis, retinal detachment, and increased intraocular pressure, can negatively and irreversibly impact the overall success of treatment. Early recognition and management of these toxicities are vital to ensuring a positive prognosis and maintaining the therapeutic benefits of anti-VEGF therapies for retinal conditions.

**Figure 4:**
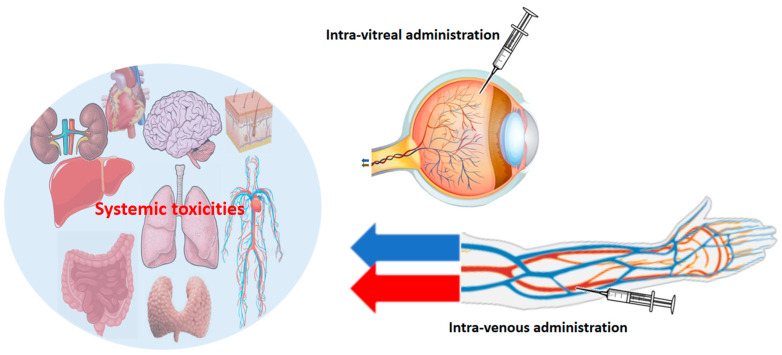
Intra-vitreal administration involves the direct injection of a substance into the vitreous humor of the eye. This route is commonly employed for the targeted delivery of VEGFi to treat ocular diseases, allowing for a concentrated and sustained release of the agent within the eye. Intra-venous administration entails the injection of the drug directly into a vein, typically entering the bloodstream, facilitating systemic drug delivery. The size of the red and blue arrows is directly and intuitively proportional to the “systemic” side effects on other organs. Systemic effects are evidently more frequent and intense when the VEGFi is administered systemically. The systemic diffusion of the drug is negligible when administered intra-vitreally (see the manuscript text for further details).

Complications arising from the systemic administration of VEGFi pose particular challenges, especially in cancer treatment. Hypertension, proteinuria, impaired wound healing, and hematologic abnormalities, including neutropenia, represent potential complications that require careful consideration. Balancing the therapeutic benefits with the risk of complications is crucial, necessitating a personalized approach to treatment. Clinicians must navigate these challenges to mitigate adverse events and optimize the effectiveness of anti-VEGF therapies, ultimately improving the prognosis for cancer patients. Complications associated with intra-vitreal administration of VEGFi, although generally localized, require careful attention. Ocular complications, such as infections and retinal issues, demand prompt intervention to preserve vision and prevent adverse outcomes. While systemic complications predominate in intra-venously or orally administered VEGFi (Figure 4), it is essential to monitor systemic complications, albeit less frequent, including the potential for thromboembolic events. Continuous refinement of strategies for minimizing complications while maximizing the therapeutic effects remains a focus in ophthalmologic practice, contributing to an improved prognosis for patients undergoing VEGFi therapy for retinal diseases.

Recognizing and managing toxicities associated with VEGFi requires expertise, experience, and a multidisciplinary approach. Multispecialty collaboration is crucial not only during the management of toxic events but also in the planning and administration phases of treatment, particularly for patients with risk factors. Moreover, the continuous refinement of strategies to minimize complications while maximizing therapeutic effects remains a consistent focus in both oncologic and ophthalmologic practices. This contributes to an improved prognosis for patients undergoing VEGFi therapy for cancer and ocular diseases.

## Figures and Tables

**Table 1:** The incidence of toxicities of all grades, as outlined by the Common Terminology Criteria for Adverse Events (CTC-AE criteria), observed with VEGFi in relation to systemic or intra-vitreal routes of administration.

Type of Toxicity	Incidence (%)	
	Intra-Venous	Intra-Vitreal
Anaemia	8	NS
Anorexia	13–58	NS
Asthenia and fatigue	50	NS
Bowel and nasal septum perforation	<0.5	NS
Brolucizumab-Associated Retinal Vasculitis	NS	0.8
Cardiac toxicity	<0.5	NS
Conjunctival hemorrhage	NS	20–40
Diarrhea	13–74	NS
Hemorrhage	<0.5	NS
Hypertension	25	NS
Hypothyroidism	10	NS
Infectious Endophthalmitis	NS	<0.001
Infusion-related hypersensitivity reactions	<0.5	NS
Intraocular inflammation	NS	<0.37
Lymphopenia	18	NS
Mucositis	4–42	NS
Nausea/vomiting	10–39	NS
Neutropenia	18	NS
Ocular Hypertension	NS	2.1–3.6
Proteinuria	8	NS
Retinal hemorrhage	NS	1–10
Reversible Posterior Leukoencephalopathy Syndrome	<0.05	NS
Rhegmatogenous retinal detachments	NS	0.013
Skin toxicity	50	NS
Thrombocytopenia	9	NS
Thromboembolic events	0.6–5.6	NS
Vitreous floaters	NS	5–15

NS: Not Significant.

**Table 2:** Overview of mechanisms underlying adverse effects induced by VEGFi.

Toxicity	Mechanism
Hypertension	Disruption of VEGF/VEGFR pathway leads to endothelial dysfunction and vasoconstriction.
Proteinuria	Inhibition of VEGF-A alters glomerular permeability, resulting in proteinuria and peripheral edema.
Thromboembolic events	Disruption of VEGF/VEGFR pathway causes endothelial cell damage.
Cardiac toxicity	VEGF-A inhibition may produce cardiomyocytes damage. Direct effects on hERG channels may cause arrhythmias.
Hemorrhage	VEGF/VEGFR pathway alteration compromises blood vessel integrity, leading to abnormal permeability and bleeding.
Bowel and nasal septum perforation	Arise from VEGFi-induced tumor necrosis or mucosal injury associated with the disruption of micro-circulation. This disruption weakens the mucosal wall, ultimately leading to arteriolar thrombosis.
Skin toxicity	Exact mechanism is unclear, hypothesized to stem from VEGF signaling disruption, affecting blood flow and vascular permeability in the skin.
Reversible Posterior Leukoencephalopathy	Mechanism likely related to VEGF pathway disruption, increasing vascular permeability at the blood-brain barrier level.
Infusion-related hypersensitivity	Exact mechanism unclear.
Hypothyroidism	Direct cytotoxic effects on thyroid follicular cells and vascular alterations in the thyroid gland.
Asthenia and fatigue	Potential causes include hypothyroidism, myocardial changes, VEGF-inhibitor-induced anorexia, and dehydration.
Gastrointestinal toxicity	VEGF inhibition diminishes blood flow to mucosal tissues, causing inflammation and damage.
Anorexia	Hypothesized to involve central nervous system interactions, potentially linked to changes in neuroendocrine signaling and appetite regulation.
Myelotoxicity	VEGFi impacts bone marrow by disrupting vascular networks and directly affecting hematopoietic stem cells, leading to diminished blood cell production.
Ocular hypertension	Anti-VEGF injections lead to transient intraocular pressure elevation, with repetitive injections linked to persistent ocular hypertension.
Intraocular inflammation	Pathogenesis unclear, may be linked to patient-specific immune responses, manufacturing impurities, and errors in provider preparation.
Brolucizumab-Associated Retinal Vasculitis	Hypotheses include severely reduced vascular perfusion and local production of anti-brolucizumab antibodies. Vasculitis affects retinal arteries.
Infectious endophthalmitis	Infection affects internal eye structures, causing inflammation and tissue destruction.

hERG: human Ether-à-go-go-Related Gene; VEGF: Vascular Endothelial Growth Factor; VEGFR: Vascular Endothelial Growth Factor Receptor; VEGF-A: Vascular Endothelial Growth Factor isoform A.

**Table 3:** Description of primary clinical manifestations associated with VEGFi toxicities.

Toxicity	Clinical Manifestation
Hypertension	Headaches, dizziness, visual disturbances, epistaxis, fatigue, shortness of breath.
Proteinuria	Peripheral edema, foamy or frothy urine, hypertension.
Thromboembolic events	Leg swelling and pain, shortness of breath, chest discomfort, coughing up blood.
Cardiac toxicity	Asthenia, fatigue, shortness of breath, fluid retention, peripheral edema, hepatomegaly, QT prolongation.
Hemorrhage	Diverse clinical presentation ranging from epistaxis to severe gastrointestinal bleeding. Intracranial hemorrhage is more severe.
Bowel/Nasal perforation	Acute abdominal pain, peritonitis, fever, nausea, vomiting, constipation or diarrhea. Nasal symptoms like epistaxis and difficulty breathing.
Skin toxicity	Hand–foot syndrome, rash, mucositis, pruritus, alopecia, subungual hemorrhage.
Reversible Posterior Leukoencephalo-pathy	Severe headache, nausea, confusion, cortical blindness, seizures.
Infusion-related reactions	Allergic symptoms to anaphylactoid reactions, flushing, itching, hypertension, wheezing, chest pain.
Hypothyroidism	Nonspecific symptoms like fatigue, unexplained weight gain, sensitivity to cold, dry skin and hair, muscle aches, depression.
Asthenia and fatigue	Reduced energy levels, physical or mental weariness.
Gastrointestinal toxicity	Nausea, vomiting, heightened salivation, pallor, mucositis, diarrhea, anorexia.
Myelotoxicity	Anemia, neutropenia, lymphopenia, thrombocytopenia with respective symptoms.
Conjunctival hemorrhage	Visible red discoloration in the conjunctiva, irritation, sensation of a foreign body in the eye.
Vitreous floaters	Perceived specks, dots, or cobweb-like shapes in the visual field, can cause visual disturbances.
Rhegmatogenous retinal detachments	Sudden onset marked by flashes of light, appearance of new or increased floaters, visual impairment.
Retinal hemorrhage	Blood within the retina, sudden changes in vision, pain or discomfort.
Ocular hypertension	Endures without discernible discomfort, routine eye examinations are imperative.
Intraocular inflammation	Pseudoendophthalmitis, acute-onset inflammation without infection, blurred vision, floaters, pain, photophobia.
Brolucizumab-Associated Retinal Vasculitis	Clinical evidence of inflammation without infectious endophthalmitis, worse visual acuity than baseline.
Infectious endophthalmitis	Decreased vision, severe pain, conjunctival injection, anterior chamber cell with hypopyon, vitritis.

**Table 4:** Methods for assessment and monitoring VEGFi toxicities.

Toxicity	Assessment and Monitoring Methods
Hypertension	Regular blood pressure assessments before and after VEGFi treatment. Patient education on self-monitoring at home.
Proteinuria	Comprehensive urinalysis test with eventual 24 h urine collection for protein evaluation.
Thromboembolic events	Patient education on recognizing signs and symptoms. Imaging techniques (ultrasound, CT) for diagnosis.
Cardiac toxicity	Periodic monitoring of QT interval. Echocardiography and routine ECG for cardiac function assessment.
Hemorrhage	Regular clinical assessments for bleeding signs. Hematological tests for platelet counts and coagulation parameters.
Bowel/Nasal septum perforation	Vigilant clinical monitoring for early detection of signs and symptoms.
Skin toxicity	Periodic clinical inspection for early signs of cutaneous toxicity.
Reversible Posterior Leu-koencephalo-pathy	Diagnostic tests include brain imaging (MRI) and blood pressure monitoring.
Infusion-related hypersensitivity reactions	Close clinical observation during and after drug infusion.
Hypothyroidism	Regular monitoring of blood biochemistry, including thyroid function.
Asthenia and fatigue	Systematic clinical examinations and specific questionnaires. Blood tests to rule out physiological causes.
Gastrointestinal toxicity	Diagnosis through clinical examination and anamnestic collection. Electrolyte tests for assessing vomiting and diarrhea.
Anorexia	Clinical assessment, BMI measurement, and blood tests for nutritional parameters. Consideration of psychological criteria.
Myelotoxicity	Combination of clinical evaluation and laboratory tests.
Conjunctival hemorrhage	Diagnosis based on clinical presentation with subconjunctival bleeding.
Vitreous floaters	Visualization techniques (fundus examination, OCT, ultrasound) for assessment.
Rhegmatogenous retinal detachments	Imaging modalities (ultrasound, OCT) for diagnosis of retinal detachments.
Retinal hemorrhage	Fundus examination, OCT, or fluorescein angiography for visualization.
Ocular hypertension	Tonometry (GAT, non-contact, rebound) for IOP evaluation. Visual field evaluation with Goldmann or automated perimetry.
Intraocular inflammation	Laboratory tests (CBC, ESR, CRP) for systemic inflammation. Instrumental tests (slit-lamp, fundus photography, OCT).
Brolucizumab-Associated Retinal Vasculitis	Fundus examination and obtaining serum and vitreous cultures. Rule out infectious endophthalmitis.
Infectious endophthalmitis	Needle-based vitreous sampling for microbiologic analysis. Inflammatory markers (ESR, CRP) and ocular imaging.

BMI: Body Mass Index; CBC: Cell Blood Count; CRP: C-reactive protein; CT: Computed tomography; ECG: electrocardiogram; ESR: erythrocyte sedimentation rate; GAT: Goldmann Applanation Tonometry; MRI: magnetic resonance imaging; OCT: optical coherence tomography.

## References

[1] Thapa K., Khan H., Kaur G., Kumar P., Singh T.G. (2023). Therapeutic targeting of angiopoietins in tumor angiogenesis and cancer development. Biochem. Biophys. Res. Commun..

[2] Patel S.A., Nilsson M.B., Le X., Cascone T., Jain R.K., Heymach J.V. (2023). Molecular Mechanisms and Future Implications of VEGF/VEGFR in Cancer Therapy. Clin. Cancer Res..

[3] Apte R.S., Chen D.S., Ferrara N. (2019). VEGF in Signaling and Disease: Beyond Discovery and Development. Cell.

[4] Brouillet S., Hoffmann P., Feige J.-J., Alfaidy N. (2012). EG-VEGF: A key endocrine factor in placental development. Trends Endocrinol. Metab..

[5] Trifanescu O.G., Gales L.N., Tanase B.C., Marinescu S.A., Trifanescu R.A., Gruia I.M., Paun M.A., Rebegea L., Mitrica R., Serbanescu L. (2023). Prognostic Role of Vascular Endothelial Growth Factor and Correlation with Oxidative Stress Markers in Locally Advanced and Metastatic Ovarian Cancer Patients. Diagnostics.

[6] Pérez-Gutiérrez L., Ferrara N. (2023). Biology and therapeutic targeting of vascular endothelial growth factor A. Nat. Rev. Mol. Cell Biol..

[7] Roskoski R. (2008). VEGF receptor protein–tyrosine kinases: Structure and regulation. Biochem. Biophys. Res. Commun..

[8] Mabeta P., Steenkamp V. (2022). The VEGF/VEGFR Axis Revisited: Implications for Cancer Therapy. Int. J. Mol. Sci..

[9] Rosen L.S., Jacobs I.A., Burkes R.L. (2017). Bevacizumab in Colorectal Cancer: Current Role in Treatment and the Potential of Biosimilars. Target. Oncol..

[10] Mukherji S.K. (2010). Bevacizumab (Avastin). Am. J. Neuroradiol..

[11] Garcia J., Hurwitz H.I., Sandler A.B., Miles D., Coleman R.L., Deurloo R., Chinot O.L. (2020). Bevacizumab (Avastin^®^) in cancer treatment: A review of 15 years of clinical experience and future outlook. Cancer Treat. Rev..

[12] Li J., Turner D.C., Li F., Chen X., Liao M.Z., Li C. (2023). Pharmacokinetics of biologics in gastric cancer. Clin. Transl. Sci..

[13] Owen J.S., Rackley R.J., Hummel M.A., Roepcke S., Huang H., Liu M., Idris T.A., Murugesan S.M.N., Marwah A., Loganathan S. (2023). Population Pharmacokinetics of MYL-1402O, a Proposed Biosimilar to Bevacizumab and Reference Product (Avastin^®^) in Patients with Non-squamous Non-small Cell Lung Cancer. Eur. J. Drug Metab. Pharmacokinet..

[14] Estarreja J., Mendes P., Silva C., Camacho P., Mateus V. (2023). The Efficacy, Safety, and Efficiency of the Off-Label Use of Bevacizumab in Patients Diagnosed with Age-Related Macular Degeneration: Protocol for a Systematic Review and Meta-Analysis. JMIR Res. Protoc..

[15] Dupuis-Girod S., Rivière S., Lavigne C., Fargeton A., Gilbert-Dussardier B., Grobost V., Leguy-Seguin V., Maillard H., Mohamed S., Decullier E. (2023). Efficacy and safety of intravenous bevacizumab on severe bleeding associated with hemorrhagic hereditary telangiectasia: A national, randomized multicenter trial. J. Intern. Med..

[16] Stewart M.W. (2013). Off-Label Drug Use: The Bevacizumab Story. Mayo Clin. Proc..

[17] Poole R.M., Vaidya A. (2014). Ramucirumab: First Global Approval. Drugs.

[18] Greig S.L., Keating G.M. (2015). Ramucirumab: A Review in Advanced Gastric Cancer: Clinical immunotherapeutics, biopharmaceuticals and gene therapy. BioDrugs.

[19] Garon E.B., Visseren-Grul C., Rizzo M.T., Puri T., Chenji S., Reck M. (2023). Clinical outcomes of ramucirumab plus docetaxel in the treatment of patients with non-small cell lung cancer after immunotherapy: A systematic literature review. Front. Oncol..

[20] Gordan J.D., Kennedy E.B., Abou-Alfa G.K., Beg M.S., Brower S.T., Gade T.P., Goff L., Gupta S., Guy J., Harris W.P. (2020). Systemic Therapy for Advanced Hepatocellular Carcinoma: ASCO Guideline. J. Clin. Oncol..

[21] Gambardella V., Tarazona N., Cejalvo J.M., Roselló S., Cervantes A. (2016). Clinical pharmacokinetics and pharmacodynamics of ramucirumab in the treatment of colorectal cancer. Expert Opin. Drug Metab. Toxicol..

[22] Sun W., Patel A. (2014). Ziv-aflibercept in metastatic colorectal cancer. Biol. Targets Ther..

[23] Ciombor K.K., Berlin J. (2014). Aflibercept—A Decoy VEGF Receptor. Curr. Oncol. Rep..

[24] Sanz-Garcia E., Saurí T., Tabernero J., Macarulla T. (2015). Pharmacokinetic and pharmacodynamic evaluation of aflibercept for the treatment of colorectal cancer. Expert Opin. Drug Metab. Toxicol..

[25] Tadayoni R., Sararols L., Weissgerber G., Verma R., Clemens A., Holz F.G. (2021). Brolucizumab: A Newly Developed Anti-VEGF Molecule for the Treatment of Neovascular Age-Related Macular Degeneration. Ophthalmologica.

[26] Abu Serhan H., Taha M.J.J., Abuawwad M.T., Abdelaal A., Irshaidat S., Abu Serhan L., Abu Salim Q.F., Awamleh N., Abdelazeem B., Elnahry A.G. (2023). Safety and Efficacy of Brolucizumab in the Treatment of Diabetic Macular Edema and Diabetic Retinopathy: A Systematic Review and Meta-Analysis. Semin. Ophthalmol..

[27] Jakubiak P., Alvarez-Sánchez R., Fueth M., Broders O., Kettenberger H., Stubenrauch K., Caruso A. (2021). Ocular Pharmacokinetics of Intravitreally Injected Protein Therapeutics: Comparison among Standard-of-Care Formats. Mol. Pharm..

[28] Veritti D., Sarao V., Gorni G., Lanzetta P. (2022). Anti-VEGF Drugs Dynamics: Relevance for Clinical Practice. Pharmaceutics.

[29] Chatziralli I. (2021). Ranibizumab for the treatment of diabetic retinopathy. Expert Opin. Biol. Ther..

[30] Dervenis N., Mikropoulou A.M., Tranos P., Dervenis P. (2017). Ranibizumab in the Treatment of Diabetic Macular Edema: A Review of the Current Status, Unmet Needs, and Emerging Challenges. Adv. Ther..

[31] García-Quintanilla L., Luaces-Rodríguez A., Gil-Martínez M., Mondelo-García C., Maroñas O., Mangas-Sanjuan V., González-Barcia M., Zarra-Ferro I., Aguiar P., Otero-Espinar F.J. (2019). Pharmacokinetics of Intravitreal Anti-VEGF Drugs in Age-Related Macular Degeneration. Pharmaceutics.

[32] Sharma K., Suresh P.S., Mullangi R., Srinivas N.R. (2015). Quantitation of VEGFR2 (vascular endothelial growth factor receptor) inhibitors—Review of assay methodologies and perspectives. Biomed. Chromatogr..

[33] Uemura A., Fruttiger M., D’Amore P.A., De Falco S., Joussen A.M., Sennlaub F., Brunck L.R., Johnson K.T., Lambrou G.N., Rittenhouse K.D. (2021). VEGFR1 signaling in retinal angiogenesis and microinflammation. Prog. Retin. Eye Res..

[34] Șandor A., Ionuț I., Marc G., Oniga I., Eniu D., Oniga O. (2023). Structure–Activity Relationship Studies Based on Quinazoline Derivatives as EGFR Kinase Inhibitors (2017–Present). Pharmaceuticals.

[35] Dorff T.B., Pal S.K., Quinn D.I. (2014). Novel tyrosine kinase inhibitors for renal cell carcinoma. Expert Rev. Clin. Pharmacol..

[36] Modi S.J., Kulkarni V.M. (2022). Exploration of structural requirements for the inhibition of VEGFR-2 tyrosine kinase: Binding site analysis of type II, ‘DFG-out’ inhibitors. J. Biomol. Struct. Dyn..

[37] Takahashi S. (2011). Vascular endothelial growth factor (VEGF), VEGF receptors and their inhibitors for antiangiogenic tumor therapy. Biol. Pharm. Bull..

[38] Hao Z., Sadek I. (2016). Sunitinib: The antiangiogenic effects and beyond. OncoTargets Ther..

[39] Nassif E., Thibault C., Vano Y., Fournier L., Mauge L., Verkarre V., Timsit M.-O., Mejean A., Tartour E., Oudard S. (2017). Sunitinib in kidney cancer: 10 years of experience and development. Expert Rev. Anticancer. Ther..

[40] Al-Ghusn A.I., Bakheit A.H., Attwa M.W., AlRabiah H. (2023). Vandetanib. Profiles of Drug Substances, Excipients, and Related Methodology.

[41] Miyamoto S., Kakutani S., Sato Y., Hanashi A., Kinoshita Y., Ishikawa A. (2018). Drug review: Pazopanib. Jpn. J. Clin. Oncol..

[42] Limvorasak S., Posadas E.M. (2009). Pazopanib: Therapeutic developments. Expert Opin. Pharmacother..

[43] Chen Y., Tortorici M.A., Garrett M., Hee B., Klamerus K.J., Pithavala Y.K. (2013). Clinical pharmacology of axitinib. Clin. Pharmacokinet..

[44] Maroto P., Porta C., Capdevila J., Apolo A.B., Viteri S., Rodriguez-Antona C., Martin L., Castellano D. (2022). Cabozantinib for the treatment of solid tumors: A systematic review. Ther. Adv. Med. Oncol..

[45] Ettrich T.J., Seufferlein T. (2018). Regorafenib. Recent results in cancer research. Fortschritte der Krebsforschung. Prog. Dans Les Rech. Sur Le Cancer.

[46] Arai H., Battaglin F., Wang J., Lo J.H., Soni S., Zhang W., Lenz H.-J. (2019). Molecular insight of regorafenib treatment for colorectal cancer. Cancer Treat. Rev..

[47] Strumberg D., Schultheis B. (2012). Regorafenib for cancer. Expert Opin. Investig. Drugs.

[48] Lamb Y.N. (2021). Nintedanib: A Review in Fibrotic Interstitial Lung Diseases. Drugs.

[49] Wind S., Schmid U., Freiwald M., Marzin K., Lotz R., Ebner T., Stopfer P., Dallinger C. (2019). Clinical Pharmacokinetics and Pharmacodynamics of Nintedanib. Clin. Pharmacokinet..

[50] Gao Y., Ding Y., Tai X.-R., Zhang C., Wang D. (2023). Ponatinib: An update on its drug targets, therapeutic potential and safety. Biochim. Biophys. Acta BBA Rev. Cancer.

[51] Motzer R.J., Taylor M.H., Evans T.R.J., Okusaka T., Glen H., Lubiniecki G.M., Dutcus C., Smith A.D., Okpara C.E., Hussein Z. (2022). Lenvatinib dose, efficacy, and safety in the treatment of multiple malignancies. Expert Rev. Anticancer Ther..

[52] Hao Z., Wang P. (2020). Lenvatinib in Management of Solid Tumors. Oncologist.

[53] Chawla P.A., Passi I., Billowria K., Kumar B. (2023). Tivozanib: A New Hope for Treating Renal Cell Carcinoma. Anticancer Agents Med. Chem..

[54] Xie C., Zhou J., Guo Z., Diao X., Gao Z., Zhong D., Jiang H., Zhang L., Chen X. (2013). Metabolism and bioactivation of famitinib, a novel inhibitor of receptor tyrosine kinase, in cancer patients. Br. J. Pharmacol..

[55] Scott L.J. (2018). Apatinib: A Review in Advanced Gastric Cancer and Other Advanced Cancers. Drugs.

[56] Geng R., Li J. (2015). Apatinib for the treatment of gastric cancer. Expert Opin. Pharmacother..

[57] Schenone S., Bondavalli F., Botta M. (2007). Antiangiogenic agents: An update on small molecule VEGFR inhibitors. Curr. Med. Chem..

[58] Chaar M., Kamta J., Ait-Oudhia S. (2018). Mechanisms, monitoring, and management of tyrosine kinase inhibitors-associated cardiovascular toxicities. OncoTargets Ther..

[59] Dembinska-Kiec A., Dulak J., Partyka L., Huk I., Mailnski T. (1997). VEGF–nitric oxide reciprocal regulation. Nat. Med..

[60] Feliers D., Chen X., Akis N., Choudhury G.G., Madaio M., Kasinath B.S. (2005). VEGF regulation of endothelial nitric oxide synthase in glomerular endothelial cells. Kidney Int..

[61] Kroll J., Waltenberger J. (1999). A novel function of VEGF receptor-2 (KDR): Rapid release of nitric oxide in response to VEGF-A stimulation in endothelial cells. Biochem. Biophys. Res. Commun..

[62] Sane D.C., Anton L., Brosnihan K.B. (2004). Angiogenic growth factors and hypertension. Angiogenesis.

[63] Greene A.S., Amaral S.L. (2002). Microvascular angiogenesis and the renin-angiotensin system. Curr. Hypertens. Rep..

[64] Byrne A.M., Bouchier-Hayes D., Harmey J. (2005). Angiogenic and cell survival functions of Vascular Endothelial Growth Factor (VEGF). J. Cell. Mol. Med..

[65] Van Hinsbergh V.W.M. (2012). Endothelium—Role in regulation of coagulation and inflammation. Semin. Immunopathol..

[66] van Hinsbergh V.W. (2001). The endothelium: Vascular control of haemostasis. European journal of obstetrics, gynecology, and reproductive biology. Eur. J. Obstet. Gynecol. Reprod. Biol..

[67] Wautier J.-L., Wautier M.-P. (2022). Vascular Permeability in Diseases. Int. J. Mol. Sci..

[68] Hu K., Olsen B.R. (2016). The roles of vascular endothelial growth factor in bone repair and regeneration. Bone.

[69] DiPietro L.A. (2016). Angiogenesis and wound repair: When enough is enough. J. Leukoc. Biol..

[70] Parmar D., Apte M. (2021). Angiopoietin inhibitors: A review on targeting tumor angiogenesis. Eur. J. Pharmacol..

[71] Cutroneo P.M., Giardina C., Ientile V., Potenza S., Sottosanti L., Ferrajolo C., Trombetta C.J., Trifirò G. (2017). Overview of the Safety of Anti-VEGF Drugs: Analysis of the Italian Spontaneous Reporting System. Drug Saf..

[72] Jiang L., Ping L., Yan H., Yang X., He Q., Xu Z., Luo P. (2020). Cardiovascular toxicity induced by anti-VEGF/VEGFR agents: A special focus on definitions, diagnoses, mechanisms and management. Expert Opin. Drug Metab. Toxicol..

[73] Papadimitriou K., Rolfo C., Dewaele E., Van De Wiel M., Brande J.V.D., Altintas S., Huizing M., Specenier P., Peeters M. (2015). Incorporating anti-VEGF pathway therapy as a continuum of care in metastatic colorectal cancer. Curr. Treat. Options Oncol..

[74] Riondino S., Del Monte G., Fratangeli F., Guadagni F., Roselli M., Ferroni P. (2017). Anti-Angiogenic Drugs, Vascular Toxicity and Thromboembolism in Solid Cancer. Cardiovasc. Hematol. Agents Med. Chem..

[75] Caraglia M., Santini D., Bronte G., Rizzo S., Sortino G., Rini G.B., Di Fede G., Russo A. (2011). Predicting Efficacy and Toxicity in the Era of Targeted Therapy: Focus on Anti-EGFR and Anti-VEGF Molecules. Curr. Drug Metab..

[76] Hayman S.R., Leung N., Grande J.P., Garovic V.D. (2012). VEGF Inhibition, Hypertension, and Renal Toxicity. Curr. Oncol. Rep..

[77] Chen H.X., Cleck J.N. (2009). Adverse effects of anticancer agents that target the VEGF pathway. Nat. Rev. Clin. Oncol..

[78] Bracha P., Moore N.A., Ciulla T.A., WuDunn D., Cantor L.B. (2018). The acute and chronic effects of intravitreal anti-vascular endothelial growth factor injections on intraocular pressure: A review. Surv. Ophthalmol..

[79] Boyer D.S., Hopkins J.J., Sorof J., Ehrlich J.S. (2013). Anti-vascular endothelial growth factor therapy for diabetic macular edema. Ther. Adv. Endocrinol. Metab..

[80] Rodrigues E.B., Farah M.E., Maia M., Penha F.M., Regatieri C., Melo G.B., Pinheiro M.M., Zanetti C.R. (2009). Therapeutic monoclonal antibodies in ophthalmology. Prog. Retin. Eye Res..

[81] Hsu S.T., Ponugoti A., Deaner J.D., Vajzovic L. (2021). Update on Retinal Drug Toxicities. Curr. Ophthalmol. Rep..

[82] Ollero M., Sahali D. (2015). Inhibition of the VEGF signalling pathway and glomerular disorders. Nephrol. Dial. Transpl..

[83] Ferroni P., Formica V., Roselli M., Guadagni F. (2010). Thromboembolic events in patients treated with anti-angiogenic drugs. Curr. Vasc. Pharmacol..

[84] Braile M., Marcella S., Cristinziano L., Galdiero M.R., Modestino L., Ferrara A.L., Varricchi G., Marone G., Loffredo S. (2020). VEGF-A in Cardiomyocytes and Heart Diseases. Int. J. Mol. Sci..

[85] Kivelä R., Hemanthakumar K.A., Vaparanta K., Robciuc M., Izumiya Y., Kidoya H., Takakura N., Peng X., Sawyer D.B., Elenius K. (2019). Endothelial Cells Regulate Physiological Cardiomyocyte Growth via VEGFR2-Mediated Paracrine Signaling. Circulation.

[86] Bagnes C., Panchuk P.N., Recondo G. (2010). Antineoplastic chemotherapy induced QTc prolongation. Curr. Drug Saf..

[87] Witchel H.J. (2011). Drug-induced hERG Block and Long QT Syndrome. Cardiovasc. Ther..

[88] Thomas S.H.L., Behr E.R. (2016). Pharmacological treatment of acquired QT prolongation and torsades de pointes. Br. J. Clin. Pharmacol..

[89] Tisdale J.E. (2016). Drug-induced QT interval prolongation and torsades de pointes: Role of the pharmacist in risk assessment, prevention and management. Can. Pharm. J..

[90] Walraven M., Witteveen P.O., Lolkema M.P.J., van Hillegersberg R., Voest E.E., Verheul H.M.W. (2011). Antiangiogenic tyrosine kinase inhibition related gastrointestinal perforations: A case report and literature review. Angiogenesis.

[91] Maeda Y., Shinohara T., Minagawa N., Kobayashi T., Koyama R., Shimada S., Tsunetoshi Y., Murayama K., Hasegawa H. (2020). A retrospective analysis of emergency surgery for cases of acute abdomen during cancer chemotherapy. Case series. Ann. Med. Surg..

[92] Roodhart J.M., Langenberg M.H., Witteveen E., Voest E.E. (2008). The Molecular Basis of Class Side Effects Due to Treatment with Inhibitors of the VEGF/VEGFR Pathway. Curr. Clin. Pharmacol..

[93] Detmar M. (2000). The role of VEGF and thrombospondins in skin angiogenesis. J. Dermatol. Sci..

[94] Tlemsani C., Mir O., Boudou-Rouquette P., Huillard O., Maley K., Ropert S., Coriat R., Goldwasser F. (2011). Posterior reversible encephalopathy syndrome induced by anti-VEGF agents. Target. Oncol..

[95] Shord S.S., Bressler L.R., Tierney L.A., Cuellar S., George A. (2009). Understanding and managing the possible adverse effects associated with bevacizumab. Am. J. Health-System Pharm..

[96] Schmidinger M. (2013). Understanding and managing toxicities of vascular endothelial growth factor (VEGF) inhibitors. Eur. J. Cancer Suppl..

[97] Arora N., Gupta A., Singh P.P. (2017). Biological agents in gastrointestinal cancers: Adverse effects and their management. J. Gastrointest. Oncol..

[98] Peters A., Schweiger U., Frühwald-Schultes B., Born J., Fehm H.L. (2002). The neuroendocrine control of glucose allocation. Exp. Clin. Endocrinol. Diabetes.

[99] Liu Y., Olsen B.R. (2014). Distinct VEGF Functions During Bone Development and Homeostasis. Arch. Immunol. Ther. Exp..

[100] Florentin J., O’Neil S.P., Ohayon L.L., Uddin A., Vasamsetti S.B., Arunkumar A., Ghosh S., Boatz J.C., Sui J., Kliment C.R. (2022). VEGF Receptor 1 Promotes Hypoxia-Induced Hematopoietic Progenitor Proliferation and Differentiation. Front. Immunol..

[101] Fons P., Herault J.P., Delesque N., Tuyaret J., Bono F., Herbert J.M. (2004). VEGF-R2 and neuropilin-1 are involved in VEGF-A-induced differentiation of human bone marrow progenitor cells. J. Cell. Physiol..

[102] Kampougeris G., Spyropoulos D., Mitropoulou A. (2013). Intraocular Pressure rise after Anti-VEGF Treatment: Prevalence, Possible Mechanisms and Correlations. J. Curr. Glaucoma Pract..

[103] Good T.J., Kimura A.E., Mandava N., Kahook M.Y. (2010). Sustained elevation of intraocular pressure after intravitreal injections of anti-VEGF agents. Br. J. Ophthalmol..

[104] Cox J.T., Eliott D., Sobrin L. (2021). Inflammatory Complications of Intravitreal Anti-VEGF Injections. J. Clin. Med..

[105] Palestine A.G., Pecen P.E. (2020). Infectious Endophthalmitis in the Current Era. Ophthalmol. Retin..

[106] Brinda B.J., Viganego F., Vo T., Dolan D., Fradley M.G. (2016). Anti-VEGF-Induced Hypertension: A Review of Pathophysiology and Treatment Options. Curr. Treat. Options Cardiovasc. Med..

[107] Ishak R.S., Aad S.A., Kyei A., Farhat F.S. (2014). Cutaneous manifestations of anti-angiogenic therapy in oncology: Review with focus on VEGF inhibitors. Crit. Rev. Oncol. Hematol..

[108] Wang P., Wang D., Meng A.B., Zhi X., Zhu P., Lu L., Tang L., Pu Y., Li X. (2022). Effects of Walking on Fatigue in Cancer Patients: A Systematic Review and Meta-analysis. Cancer Nurs..

[109] Baumal C.R., Spaide R.F., Vajzovic L., Freund K.B., Walter S.D., John V., Rich R., Chaudhry N., Lakhanpal R.R., Oellers P.R. (2020). Retinal Vasculitis and Intraocular Inflammation after Intravitreal Injection of Brolucizumab. Ophthalmology.

[110] van Dorst D.C., Dobbin S.J., Neves K.B., Herrmann J., Herrmann S.M., Versmissen J., Mathijssen R.H., Danser A.J., Lang N.N. (2021). Hypertension and Prohypertensive Antineoplastic Therapies in Cancer Patients. Circ. Res..

[111] Schiffer M., Zukovic L., Hall S., Merl M.Y. (2021). Assessment of extended urine protein monitoring frequency in patients receiving bevacizumab. J. Oncol. Pharm. Pract..

[112] Raskob G.E., Silverstein R., Bratzler D.W., Heit J.A., White R.H. (2010). Surveillance for deep vein thrombosis and pulmonary embolism: Recommendations from a national workshop. Am. J. Prev. Med..

[113] Santoni M., Guerra F., Conti A., Lucarelli A., Rinaldi S., Belvederesi L., Capucci A., Berardi R. (2017). Incidence and risk of cardiotoxicity in cancer patients treated with targeted therapies. Cancer Treat. Rev..

[114] Machado M.O., Kang N.C., Tai F., Sambhi R.D.S., Berk M., Carvalho A.F., Chada L.P., Merola J.F., Piguet V., Alavi A. (2021). Measuring fatigue: A meta-review. Int. J. Dermatol..

[115] Russell M.K. (2015). Functional Assessment of Nutrition Status. Nutr. Clin. Pract..

[116] Taberna D.J., Navas-Carretero S., Martinez J.A. (2019). Current nutritional status assessment tools for metabolic care and clinical nutrition. Curr. Opin. Clin. Nutr. Metab. Care.

[117] Stewart M.W., Browning D.J., Landers M.B. (2018). Current management of diabetic tractional retinal detachments. Indian J. Ophthalmol..

[118] Pearce J.G., Maddess T. (2019). The Clinical Interpretation of Changes in Intraocular Pressure Measurements Using Goldmann Applanation Tonometry: A Review. J. Glaucoma.

[119] Baddam D.O., Ragi S.D., Tsang S.H., Ngo W.K. (2023). Ophthalmic Fluorescein Angiography. Methods Mol. Biol..

[120] Hamnvik O.R., Choueiri T.K., Turchin A., McKay R.R., Goyal L., Davis M., Kaymakcalan M.D., Williams J.S. (2015). Clinical risk factors for the development of hypertension in patients treated with inhibitors of the VEGF signaling pathway. Cancer.

[121] Moriyama S., Hieda M., Kisanuki M., Kawano S., Yokoyama T., Fukata M., Kusaba H., Maruyama T., Baba E., Akashi K. (2022). Effect of renin–angiotensin system inhibitors in patients with cancer treated with anti-VEGF therapy. Open Heart.

[122] Koskina L., Andrikou I., Thomopoulos C., Tsioufis K. (2023). Preexisting hypertension and cancer therapy: Evidence, pathophysiology, and management recommendation. J. Hum. Hypertens..

[123] Zhu X., Wu S., Dahut W.L., Parikh C.R. (2007). Risks of proteinuria and hypertension with bevacizumab, an antibody against vascular endothelial growth factor: Systematic review and meta-analysis. Am. J. Kidney Dis..

[124] Faruque L.I., Lin M., Battistella M., Wiebe N., Reiman T., Hemmelgarn B., Thomas C., Tonelli M. (2014). Systematic review of the risk of adverse outcomes associated with vascular endothelial growth factor inhibitors for the treatment of cancer. PLoS ONE.

[125] Abbas A., Mirza M.M., Ganti A.K., Tendulkar K. (2015). Renal Toxicities of Targeted Therapies. Target. Oncol..

[126] Tesařová P., Tesař V. (2013). Proteinuria and hypertension in patients treated with inhibitors of the VEGF signalling pathway-incidence, mechanisms and management. Folia Biol..

[127] Meilhac A., Cautela J., Thuny F. (2022). Cancer Therapies and Vascular Toxicities. Curr. Treat. Options Oncol..

[128] Watson N., Al-Samkari H. (2021). Thrombotic and bleeding risk of angiogenesis inhibitors in patients with and without malignancy. J. Thromb. Haemost..

[129] Touyz R.M., Herrmann S.M., Herrmann J. (2018). Vascular toxicities with VEGF inhibitor therapies–focus on hypertension and arterial thrombotic events. J. Am. Soc. Hypertens..

[130] Sundararajan S., Kumar A., Poongkunran M., Kannan A., Vogelzang N.J., Reck M., Heigener D., Reinmuth N., Sullivan I., Planchard D. (2016). Cardiovascular adverse effects of targeted antiangiogenic drugs: Mechanisms and management. Future Oncol..

[131] Mihalcea D., Memis H., Mihaila S., Vinereanu D. (2023). Cardiovascular Toxicity Induced by Vascular Endothelial Growth Factor Inhibitors. Life.

[132] Dobbin S.J., Petrie M.C., Myles R.C., Touyz R.M., Lang N.N. (2021). Cardiotoxic effects of angiogenesis inhibitors. Clin. Sci..

[133] Sandler A., Hirsh V., Reck M., von Pawel J., Akerley W., Johnson D.H. (2012). An evidence-based review of the incidence of CNS bleeding with anti-VEGF therapy in non-small cell lung cancer patients with brain metastases. Lung Cancer.

[134] Zuo P.-Y., Chen X.-L., Liu Y.-W., Xiao C.-L., Liu C.-Y. (2014). Increased risk of cerebrovascular events in patients with cancer treated with bevacizumab: A meta-analysis. PLoS ONE.

[135] McLellan B., Ciardiello F., Lacouture M.E., Segaert S., Van Cutsem E. (2015). Regorafenib-associated hand–foot skin reaction: Practical advice on diagnosis, prevention, and management. Ann. Oncol..

[136] Manchen E., Robert C., Porta C. (2011). Management of tyrosine kinase inhibitor-induced hand-foot skin reaction: Viewpoints from the medical oncologist, dermatologist, and oncology nurse. J. Support. Oncol..

[137] Sugita K., Kawakami K., Yokokawa T., Mae Y., Toya W., Hagino A., Suzuki K., Suenaga M., Mizunuma N., Yamaguchi T. (2015). Investigation of Regorafenib-induced Hypothyroidism in Patients with Metastatic Colorectal Cancer. Anticancer Res..

[138] Fernández A.A., Martín P., Martínez M.I., Bustillo M.A., Hernández F.J.B., Labrado J.d.l.C., Peñas R.D.-D., Rivas E.G., Delgado C.P., Redondo J.R. (2009). Chronic fatigue syndrome: Aetiology, diagnosis and treatment. BMC Psychiatry.

[139] Eisen T., Sternberg C.N., Robert C., Mulders P., Pyle L., Zbinden S., Izzedine H., Escudier B. (2012). Targeted Therapies for renal cell carcinoma: Review of adverse event management strategies. JNCI J. Natl. Cancer Inst..

[140] Hartmann J.T., Haap M., Kopp H.G., Lipp H.P. (2009). Tyrosine kinase inhibitors—A review on pharmacology, metabolism and side effects. Curr. Drug Metab..

[141] Fachi M.M., Tonin F.S., Leonart L.P., Rotta I., Fernandez-Llimos F., Pontarolo R. (2019). Haematological adverse events associated with tyrosine kinase inhibitors in chronic myeloid leukaemia: A network meta-analysis. Br. J. Clin. Pharmacol..

[142] Falavarjani K.G., Nguyen Q.D. (2013). Adverse events and complications associated with intravitreal injection of anti-VEGF agents: A review of literature. Eye.

[143] Van der Reis M.I., La Heij E.C., De Jong-Hesse Y., Ringens P.J., Hendrikse F., Schouten J.S.A.G. (2011). A systematic review of the adverse events of intravitreal anti-vascular endothelial growth factor injections. Retina.

[144] Tolentino M. (2011). Systemic and ocular safety of intravitreal anti-vegf therapies for ocular neovascular disease. Surv. Ophthalmol..

[145] Day S., Acquah K., Mruthyunjaya P., Grossman D.S., Lee P.P., Sloan F.A. (2011). Ocular complications after anti–vascular endothelial growth factor therapy in medicare patients with age-related macular degeneration. Arch. Ophthalmol..

[146] Daka Q., Špegel N., Velkovska M.A., Steblovnik T., Kolko M., Neziri B., Cvenkel B. (2023). Exploring the Relationship between Anti-VEGF Therapy and Glaucoma: Implications for Management Strategies. J. Clin. Med..

[147] Singh R.S.J., Kim J.E. (2012). Ocular hypertension following intravitreal anti-vascular endothelial growth factor agents. Drugs Aging.

[148] Baumal C.R., Bodaghi B., Singer M., Tanzer D.J., Seres A., Joshi M.R., Feltgen N., Gale R. (2021). Expert Opinion on Management of Intraocular Inflammation, Retinal Vasculitis, and Vascular Occlusion after Brolucizumab Treatment. Ophthalmol. Retina..

[149] Shao E.H., Yates W.B., Ho I.-V., Chang A.A., Simunovic M.P. (2021). Endophthalmitis: Changes in Presentation, Management and the Role of Early Vitrectomy. Ophthalmol. Ther..

